# Cooperativity in the assembly of H-bonded duplexes of synthetic recognition-encoded melamine oligomers†

**DOI:** 10.1039/d4sc08591d

**Published:** 2025-03-07

**Authors:** Mohit Dhiman, Luis Escobar, Joseph T. Smith, Christopher A. Hunter

**Affiliations:** a Yusuf Hamied Department of Chemistry, University of Cambridge Lensfield Road Cambridge CB2 1EW UK herchelsmith.orgchem@ch.cam.ac.uk

## Abstract

Recognition-encoded melamine oligomers (REMO) are synthetic polymers composed of repeating triazine–piperazine units and equipped with phenol and phosphine oxide side-chains. Short oligomers have previously been shown to form length- and sequence-selective H-bonded duplexes in non-polar solvents. Here, automated solid phase synthesis was used to prepare homo-sequence REMO with either twelve phenol recognition units or twelve phosphine oxide recognition units. The ends of the oligomers were functionalised with an azide and an alkyne group to allow investigation of duplex formation by covalent trapping with copper-catalysed azide–alkyne cycloaddition (CuAAC) reactions. The oligomers were also functionalised with a dansyl fluorophore or a dabcyl quencher dye to allow investigation of duplex formation by Förster resonance energy transfer (FRET). Covalent trapping showed that the duplex is the major species present in a 1 : 1 mixture of the phenol 12-mer and phosphine oxide 12-mer at micromolar concentrations in dichloromethane. FRET titration experiments showed that the association constant for duplex formation is greater than 10^8^ M^−1^ in chloroform, and DMSO denaturation experiments showed that duplex formation is highly cooperative. The Hill coefficient for denaturation of the 12-mer duplex was 4.6, which is significantly higher than the value measured for the corresponding 6-mer duplex (1.9). This behaviour mirrors that observed for nucleic acid duplexes, where denaturation becomes increasingly cooperative as more base-pairs are added to the duplex.

## Abstract 2

Fluorescence experiments (FRET) show that synthetic sequence-complementary polymers (REMO) form H-bonded duplexes in a highly cooperative all-or-nothing process that mirrors the behaviour of the DNA double helix.

## Graphical abstract

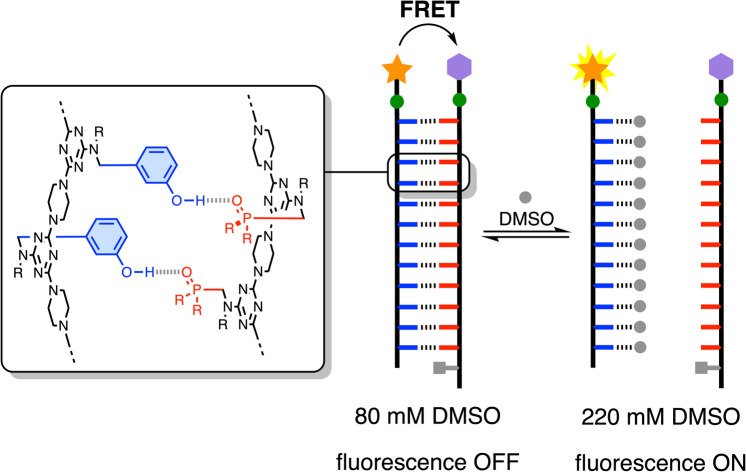

## Introduction

In nature, the structures of the key biomolecules involved in processes such as catalysis, recognition and self-assembly are linear oligomers, where the functional properties are encoded in the sequence of the monomer building blocks.^1^ Synthetic polymers made of two or more different monomers should be able to be programmed in a similar manner, but designing and characterising such systems remains a challenging and relatively unexplored area of chemistry. Synthetic chemists have drawn inspiration from the ladder motif found in the DNA double helix due to the relative simplicity of the supramolecular structure and the fact that it forms the basis for template-directed molecular replication.^2^ There are many examples of synthetic systems that form duplexes with base-pairs formed by H-bonds,^3–9^ salt bridges,^10,11^ metal–ligand coordination^12,13^ and dynamic covalent bonds.^14–16^

**Fig. 1:**
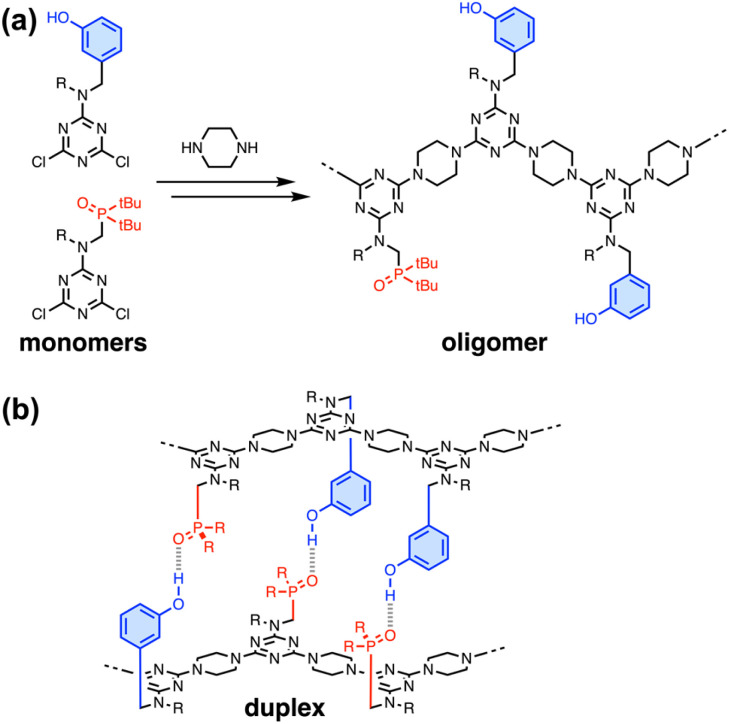
(a) Recognition-encoded melamine oligomers (REMO) are synthesised from dichlorotriazine monomers using S_N_Ar reactions with piperazine. (b) Phenol·phosphine oxide H-bonding interactions lead to the assembly of duplexes between sequence-complementary strands. R = isobutyl or 2-ethylhexyl.

Fig. 1 illustrates duplex formation by recognition-encoded melamine oligomers (REMO), which are synthetic polymers composed of a uniform backbone of repeating triazine–piperazine units and side-chains that encode information as a sequence of phenol H-bond donor and phosphine oxide H-bond acceptor recognition units.^17^ The self-assembly of REMO has previously been investigated using ITC and NMR titrations, ^31^P NMR denaturation experiments, and covalent trapping studies in non-polar solvent.

The formation of duplexes by complementary homo-oligomers has been studied for REMO up to six monomer units long, and the sequence-selectivity of duplex formation has been demonstrated by the trapping of H-bonded duplexes from libraries of hexamer sequences.^18^ Cooperative self-assembly with sharp all-or-nothing transitions between ordered and disassembled species is one of the hallmarks of biological systems. For nucleic acids, melting of the double helix occurs over progressively narrower temperature ranges as the length of the oligomers increases and the process becomes more cooperative.^19,20^ Here we investigate the effect of oligomer length on the cooperativity of duplex formation by synthetic REMO and show that complementary 12-mers form very stable duplexes with highly cooperative denaturation transitions.

## Results and discussion

### Synthesis of REMO

**Fig. 2:**
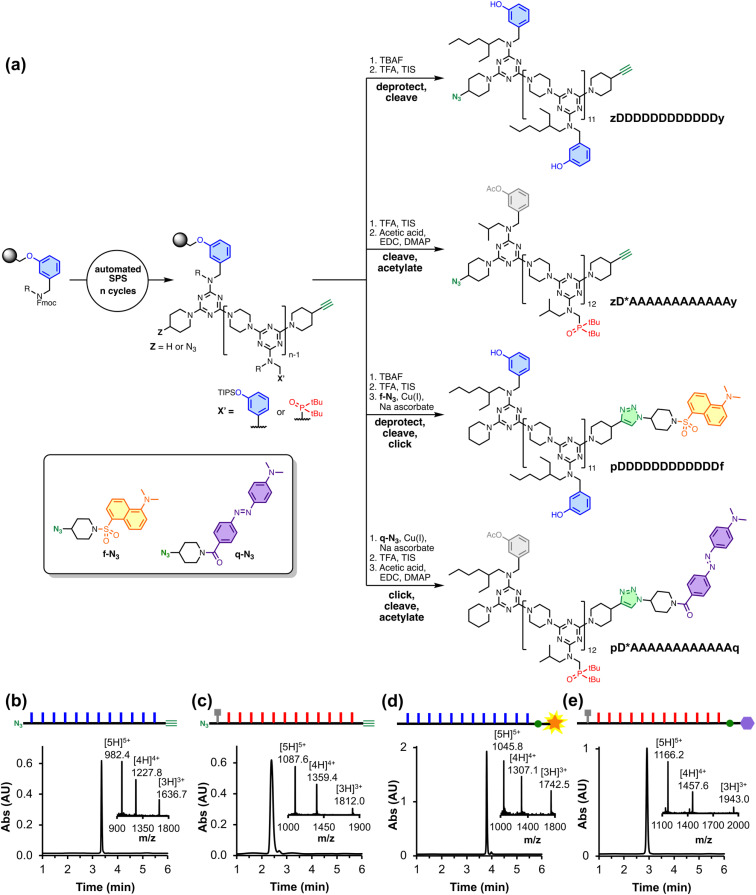
(a) REMO synthesised by SPS and then functionalised with different terminal groups. UPLC trace and ESI-MS of: (b) zDDDDDDDDDDDDy (calculated mass (ESI^+^): 1636.8 [M + 3H]^3+^, 1227.8 [M + 4H]^4+^, 982.5 [M + 5H]^5+^), (c) zD*AAAAAAAAAAAAy (calculated mass (ESI^+^): 1812.2 [M + 3H]^3+^, 1359.4 [M + 4H]^4+^, 1087.7 [M + 5H]^5+^), (d) pDDDDDDDDDDDDf (calculated mass ESI^+^: 1742.5 [M + 3H]^3+^, 1307.1 [M + 4H]^4+^, 1045.9 [M + 5H]^5+^) and (e) pD*AAAAAAAAAAAAq (calculated mass ESI^+^: 1943.0 [M + 3H]^3+^, 1457.6 [M + 4H]^4+^, 1166.2 [M + 5H]^5+^) UPLC conditions: C4 column at 40 °C using a 30–100% gradient of THF/formic acid (0.1%) in water/formic acid (0.1%) over 4 minutes, then 100% THF/formic acid (0.1%) over 2 minutes.

REMO homo-sequences were prepared by stepwise, automated solid-phase synthesis on a functionalised TentaGel Wang resin following a methodology we previously reported.^21^ Oligomers were assembled by iterative rounds of S_N_Ar reactions, alternately coupling with a dichlorotriazine monomer then piperazine. Fig. 2(a) shows the route to the four polymers utilised in this study, where the sequences are described using upper-case letters for the recognition units (D for phenol, A for phosphine oxide, and D* for acetylated phenol) and lower-case letters for the end groups (z for azide, y for alkyne, and p for piperidine). zDDDDDDDDDDDDy was synthesised directly by SPS, followed by the deprotection of the phenol groups and cleavage from the resin. The SPS route uses a terminal phenol unit to grow oligomers on the resin, which means that the first recognition unit in the sequence is always a phenol. Hence, the all-acceptor homo-oligomer zD*AAAAAAAAAAAAy was accessed by first synthesising zDAAAAAAAAAAAAy and then acetylating the terminal phenol group to prevent it from acting as a H-bond donor. The resulting acetate ester is a weak H-bond acceptor (H-bond acceptor parameter *β* = 5) and does not compete with the phosphine oxide recognition units (*β* = 11) for H-bonding interactions with the complementary phenol oligomer.^22–24^

Dansyl fluorophore and dabcyl quencher moieties were added to the ends of REMO bearing terminal alkyne groups by utilising the corresponding azide derivative, f-N_3_ or q-N_3_ (see ESI† for synthetic procedures), in copper(i)-catalysed azide–alkyne cycloaddition (CuAAC) reactions. The solution-phase CuAAC reaction of f-N_3_ with the phenol 12-mer yielded pDDDDDDDDDDDDf. pD*AAAAAAAAAAAAq was accessed by the on-resin CuAAC reaction of q-N_3_ with the phosphine oxide 12-mer, followed by cleavage of the product from the resin and acetylation of the terminal phenol group.

The UPLC traces and mass spectra in Fig. 2(b–e) show that, following preparative HPLC, all four products were obtained with a purity of greater than 97%. The oligomers were further characterised by ^1^H and ^31^P NMR, and HRMS (see ESI†).

### NMR spectroscopy

**Fig. 3:**
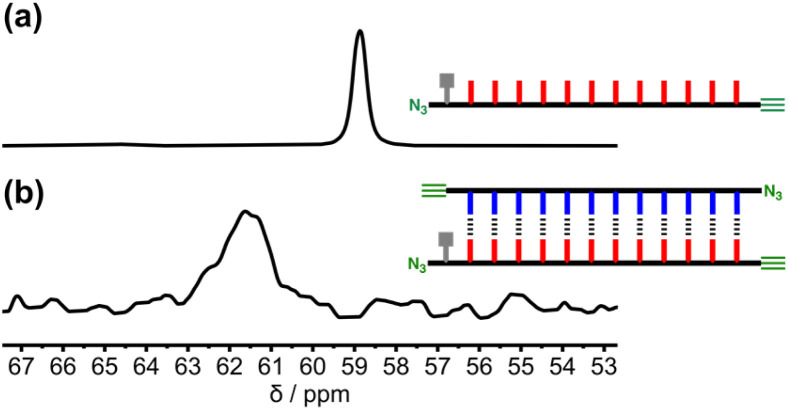
Partial ^31^P NMR spectra (162 MHz, CDCl_3_, 298 K) of (a) a 1 mM solution of zD*AAAAAAAAAAAAy and (b) a 1 mM solution of zD*AAAAAAAAAAAAy after addition of one equivalent of zDDDDDDDDDDDDy. Line broadening of 50 Hz was applied to both spectra.

REMO duplex formation has previously been studied using ^31^P NMR titrations and ITC experiments.^17^ Where the solubility was too low to measure association constants by titrations, duplex formation was studied by dissolving 1 : 1 mixtures of sequence-complementary oligomers and monitoring DMSO denaturation of the duplex by NMR spectroscopy.^18^ The ^31^P NMR spectrum of a 1 mM solution of zD*AAAAAAAAAAAAy in CDCl_3_ is shown in Fig. 3(a). The signals due to the phosphine oxides appear as one broad peak at 58.9 ppm. zDDDDDDDDDDDDy proved difficult to dissolve in non-polar solvents, but a 1 : 1 mixture of zD*AAAAAAAAAAAAy and zDDDDDDDDDDDDy proved sufficiently soluble to observe a broad signal at 61.7 ppm in the ^31^P NMR spectrum (Fig. 3(b)). The large increase in chemical shift observed in the mixture is characteristic of H-bonding interactions between the phosphine oxides and the complementary phenol recognition units, suggesting formation of the zD*AAAAAAAAAAAAy·zDDDDDDDDDDDDy duplex in which all of the phosphine oxide groups are involved in intermolecular base-pairing interactions with phenols. However, the solubility of the mixture was too poor to carry out denaturation experiments at the required millimolar concentrations.

### Covalent trapping

**Fig. 4:**
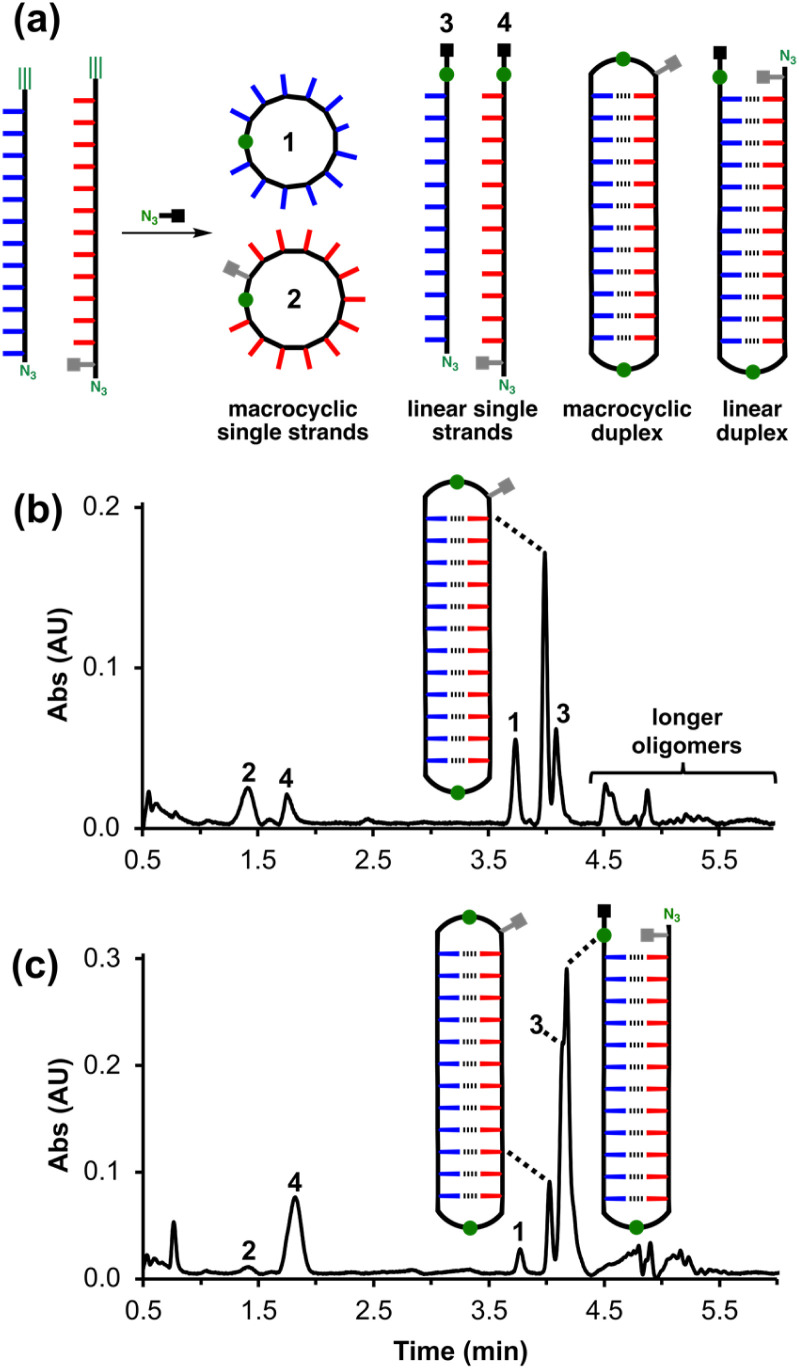
(a) Schematic representation of products formed after the CuAAC reaction of a mixture of zDDDDDDDDDDDDy and zD*AAAAAAAAAAAAy in the presence of a competing azide (green circles represent triazoles). A linear duplex could also be formed with the azide on the end of the zDDDDDDDDDDDDy chain rather than the zD*AAAAAAAAAAAAy chain (as drawn). (b) UPLC trace after reaction of zDDDDDDDDDDDDy (50 μM) and zD*AAAAAAAAAAAAy (50 μM), 4-*t*-butylbenzyl azide (100 μM) and Cu(MeCN)_4_PF_6_–TBTA (0.4 mM) in DCM at r.t. for 48 h. (c) UPLC trace after reaction of zDDDDDDDDDDDDy (50 μM) and zD*AAAAAAAAAAAAy (50 μM), 4-*t*-butylbenzyl azide (5 mM) and Cu(MeCN)_4_PF_6_–TBTA (0.4 mM) in DCM at r.t. for 48 h. The UPLC peaks were assigned based on the masses observed in the corresponding ESI-MS. UPLC conditions: C4 column at 40 °C using a 55–80% gradient of THF/formic acid (0.1%) in water/formic acid (0.1%) over 5 minutes, then up to 100% THF/formic acid (0.1%) over 1 minute.

Since zDDDDDDDDDDDDy and zD*AAAAAAAAAAAAy were synthesised with terminal azide and alkyne groups, it was possible to utilise CuAAC reactions to covalently trap the supramolecular assemblies present in dichloromethane solution. Compared to the millimolar concentrations required for NMR experiments, covalent trapping can be carried out at much lower concentrations (μM), where it is possible to ensure that the oligomers are fully dissolved. Fig. 4(a) shows cartoon representations of the different products which can form when a mixture of zDDDDDDDDDDDDy and zD*AAAAAAAAAAAAy is reacted under CuAAC conditions in the presence of another competing azide.^18,25^ If the two complementary oligomers assemble as the H-bonded duplex in a head-to-tail orientation such that the alkyne of one strand is in close proximity to the azide of the other, then an intramolecular CuAAC reaction at both ends of the duplex would lead to the macrocyclic duplex product. If the REMO backbone is sufficiently flexible, an intramolecular CuAAC reaction between the alkyne of one strand and the azide of the same strand is also possible, and this reaction would lead to macrocyclic single-stranded products. Indeed, a control experiment with the single component zD*AAAAAAAAAAAAy gave near quantitative formation of the macrocyclic single strand under CuAAC conditions (see Fig. S15†). When the reaction is carried out in the presence of 4-*t*-butylbenzylazide, a competing intermolecular process is introduced. If the concentration of 4-*t*-butylbenzylazide is greater than the effective molarity for the intramolecular reaction, then the alkyne of an oligomer will react with the competing azide, giving rise to the linear single-stranded and linear duplex products shown in Fig. 4(a).

Fig. 4(b) shows the UPLC trace after the CuAAC reaction of a 1 : 1 mixture of zDDDDDDDDDDDDy and zD*AAAAAAAAAAAAy (50 μM) in the presence of 100 μM 4-*t*-butylbenzylazide in dichloromethane. Excess competing azide and long reaction times were used to ensure complete reaction of the alkynes.^26^ The macrocyclic duplex was the major product, suggesting that the zDDDDDDDDDDDDy·zD*AAAAAAAAAAAAy duplex is the predominant species present at micromolar concentrations. Small amounts of the two macrocyclic single-stranded species and the two linear single-stranded species were observed. Some longer oligomeric products were also formed, presumably due to additional intermolecular reactions. However, no homodimeric products arising from reaction of two non-complementary oligomers were observed. The effective molarities for the intramolecular reactions that lead to the macrocyclic duplex must be much higher than 100 μM, since this species was not intercepted at 100 μM of competing azide. The duplex could assemble in the head-to-tail orientation, placing the azides and alkynes in close proximity as shown in Fig. 3(b), or in the head-to-head orientation, which would hold the azide and alkyne groups apart. The fact that the macrocyclic duplex was obtained as the major product in Fig. 4(b) suggests that the isomeric duplexes can interconvert on the timescale of the trapping reaction.

Fig. 4(c) shows that when the trapping reaction was repeated in the presence of a large excess of 4-*t*-butylbenzylazide azide (5 mM), the amounts of all three macrocyclic products formed were significantly reduced, and the major products were the linear duplex and the linear single strands. The presence of similar amounts of linear duplex and linear single-stranded products indicates that the effective molarity for the first intramolecular CuAAC reaction in the duplex is similar to the competing azide concentration (5 mM). The persistence of duplex products as the major species in both Fig. 4(b and c) provides strong evidence for the assembly of zDDDDDDDDDDDDy and zD*AAAAAAAAAAAAy as a H-bonded duplex in dichloromethane solution at micromolar concentrations.

### FRET experiments

Förster resonance energy transfer (FRET) experiments were employed to further investigate the duplex formation of 12-mer homo-oligomers. The fluorescence quenching efficiency is inversely proportional to the sixth power of the distance between fluorophore and quencher,^27^ so FRET can be used to investigate the proximity of two dyes.^28–33^ By appending one oligomer with a fluorescent group, and a sequence-complementary oligomer with a fluorescence quencher, it is possible to use changes in the fluorescence intensity to monitor duplex formation, which brings the fluorophore and quencher physically close in space. Dansyl and dabcyl were chosen as a fluorophore–quencher pair. The efficient FRET quenching of the dansyl–dabcyl pair has been demonstrated previously, since the fluorescent emission of the dansyl motif exhibits good spectral overlap with the UV-vis absorption of dabcyl.^32,33^ Neither chromophore contains functional groups that can compete for H-bonding interactions with the REMO recognition groups: sulfonamide is a weaker H-bond donor than phenol, and a weaker H-bond acceptor than phosphine oxide.^34^

**Fig. 5:**
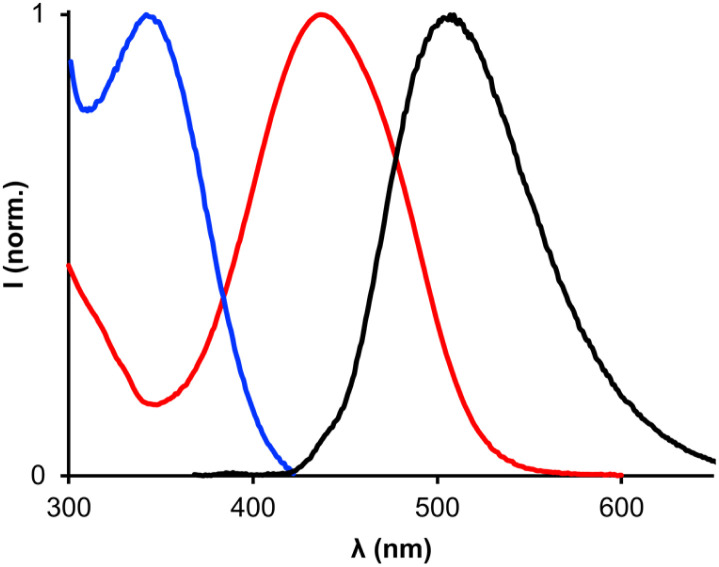
Normalised UV/vis absorption spectra of 50 μM pDDDDDDDDDDDDf (blue line) and 50 μM pD*AAAAAAAAAAAAq (red line), and the fluorescence emission spectrum (*λ*_ex_ = 348 nm) of 0.5 μM pDDDDDDDDDDDDf (black line). All spectra were recorded in chloroform at 298 K.

Fig. 5 shows the UV-vis absorption spectrum of pDDDDDDDDDDDDf in chloroform (blue line), where there is a band with a maximum at *λ*_abs,max_ = 341 nm. Excitation of pDDDDDDDDDDDDf at 348 nm produces a fluorescence emission spectrum with a maximum at *λ*_em,max_ = 505 nm which is characteristic of the dansyl group (Fig. 5, black line). The UV-vis absorption spectrum of pD*AAAAAAAAAAAAq shows an absorption maximum at *λ*_abs,max_ = 437 nm (Fig. 5, red line). There is significant spectral overlap between the pDDDDDDDDDDDDf fluorescence emission and the pD*AAAAAAAAAAAAq absorption which should lead to fluorescence quenching when the fluorophore and quencher are in close proximity.

**Fig. 6:**
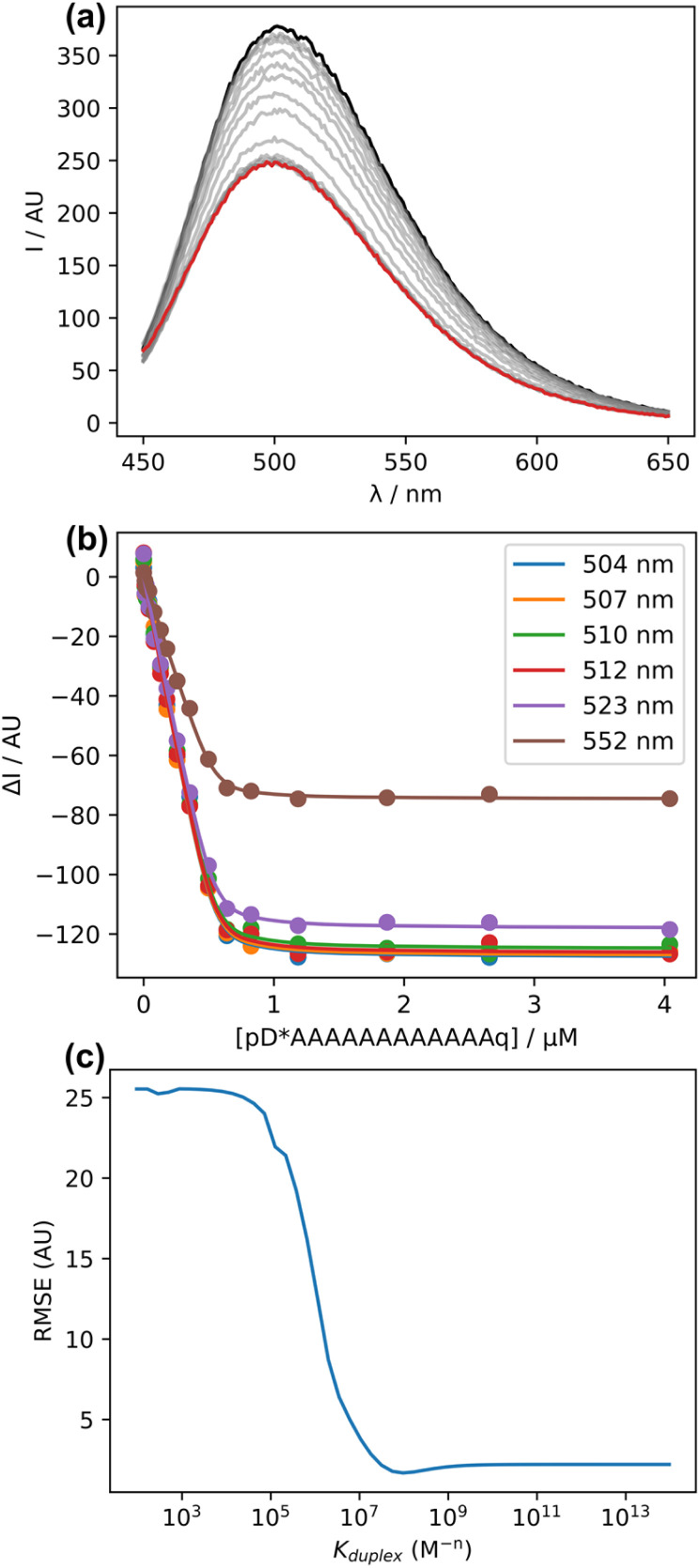
(a) Fluorescence emission spectra (*λ*_ex_ = 348 nm) for the titration of pD*AAAAAAAAAAAAq into pDDDDDDDDDDDDf (0.5 μM) in chloroform at 298 K. The fluorescence spectrum of pDDDDDDDDDDDDf and the final point of the titration are shown in black and red respectively. (b) Change in fluorescence intensity as a function of guest concentration. The lines show the best fit at six different wavelengths to a 1 : 1 binding isotherm with *K*_duplex_ = 9 × 10^7^ M^−1^. (c) The root mean squared error (RMSE) between the calculated and experimental spectra for different values of *K*_duplex_.

Fig. 6(a) shows the fluorescence emission spectra recorded after additions of pD*AAAAAAAAAAAAq to a 0.5 μM solution of pDDDDDDDDDDDDf in chloroform. As pD*AAAAAAAAAAAAq was added, there was a decrease in the intensity of the emission peak at 505 nm. The decrease in intensity of the pDDDDDDDDDDDDf emission is attributed to FRET quenching due to the formation of a duplex between the sequence-complementary oligomers. Fig. 6(b) shows the change in fluorescence intensity as a function of the guest concentration and the best fit of the data at six different wavelengths to a 1 : 1 binding isotherm, fitted using the open-source Musketeer software.^35^ The shape of the binding isotherm indicates that the system is close to the tight binding limit, and the root mean square error (RMSE) between the calculated and experimental spectra is similar for all values of association constant (*K*_duplex_) greater than the optimised value of 9 × 10^7^ M^−1^ (Fig. 6(c)). We can conclude that the association constant for formation of the 12-mer duplex in chloroform is greater than 10^8^ M^−1^, which is significantly higher than the value of 10^7^ M^−1^ measured previously for a 6-mer duplex in the less polar solvent 1,1,2,2-tetrachloroethane.^18^

The association constant for assembly of a duplex with *N* base-pairs, *K*_*N*_, depends on the strength of the base-pairing interactions, which is given by *K*_1_, the association constant for formation of one base-pair, and the effective molarities for formation of the intramolecular base-pairing interactions that zip up the duplex, EM. Assuming that the value of EM does not change significantly with the length of the duplex, the association constant for duplex formation can be estimated using eqn (1).

1 *K*_*N*_ = 2*K*_1_^*N*^EM^*N*−1^

where the statistical factor of two accounts for the degeneracy of the head-to-head and head-to-tail duplexes formed by homo-oligomers.

We have previously investigated the assembly of shorter oligomers in toluene and 1,1,2,2-tetrachlorethane, which gave values of EM in the range 40–80 mM for REMO duplexes.^17,18^ The association constant for formation of a single phenol·phosphine oxide H-bond was measured in chloroform, *K*_1_ = 120 ± 20 M^−1^ (Fig. S19†). Using these values in eqn (1) gives a value of *K*_12_ in the range 10^10^–10^13^ M^−1^ for assembly of the pDDDDDDDDDDDDf·pD*AAAAAAAAAAAAq duplex in chloroform, which is consistent with the titration data in Fig. 6.

The fluorescence intensity measured for the fully bound duplex in Fig. 6(a) is about half that of the single stranded oligomer pDDDDDDDDDDDDf. The duplex can adopt two different orientations, one which places the two chromophores in close proximity on the same end, and one which places the chromophores at opposite ends of the duplex. It is possible that quenching is only efficient in the first orientation, which might account for the residual fluorescence observed for the duplex.

**Fig. 7:**
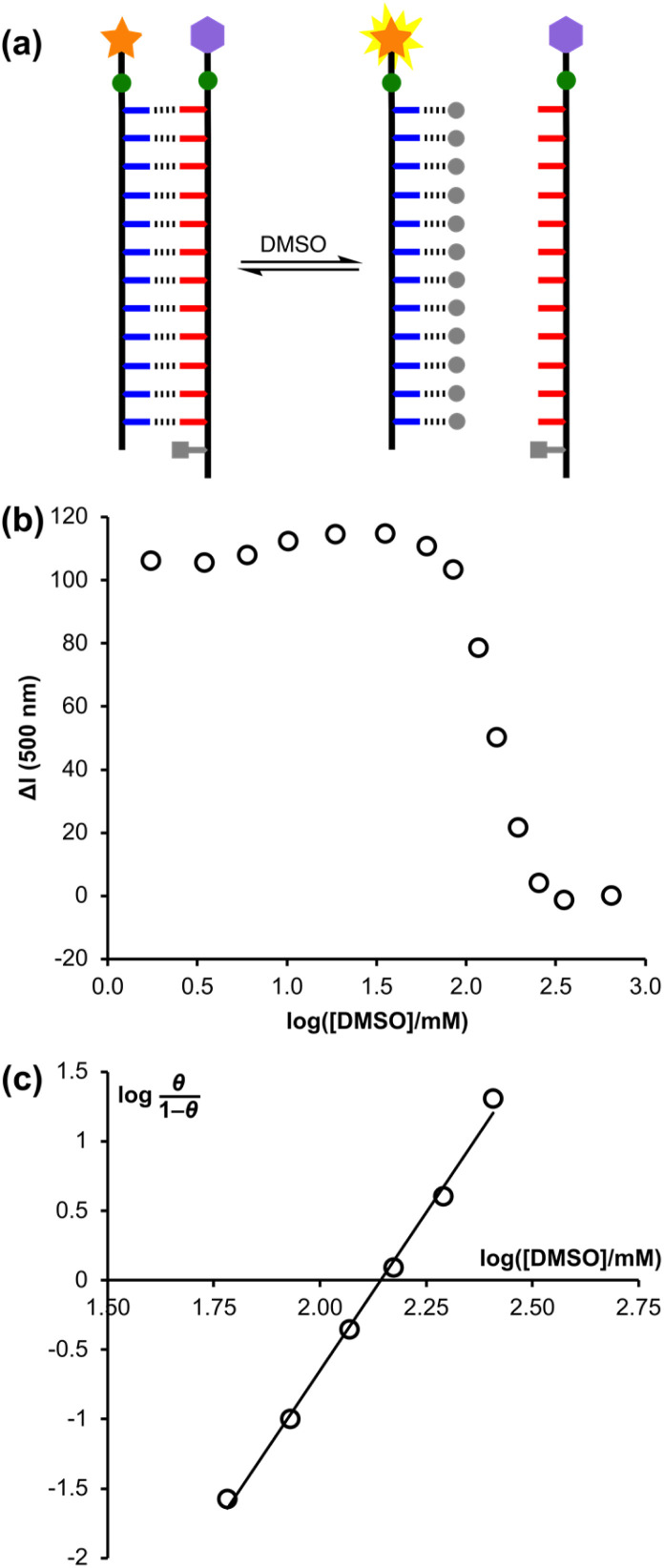
(a) DMSO denaturation of the pDDDDDDDDDDDDf·pD*AAAAAAAAAAAAq duplex (DMSO shown as grey circles). (b) Change in fluorescence intensity at 500 nm for the denaturation experiment plotted as a function of DMSO concentration in chloroform at 298 K. Δ*I* is the DMSO-induced change in fluorescence intensity of the 1 : 1 pDDDDDDDDDDDDf·pD*AAAAAAAAAAAAq mixture relative to the change in fluorescence intensity observed for pDDDDDDDDDDDDf alone at the same concentration (see ESI† for details). (c) Hill plot for the denaturation experiment showing the best fit straight line, *y* = 4.6*x* − 9.8.

Addition of competitors such as DMSO can be used to denature H-bonded duplexes, since interactions between DMSO and the phenol recognition units compete with duplex formation (Fig. 7(a)).^7^ A denaturation experiment was carried out to confirm that the decrease in fluorescence intensity seen in Fig. 6(b) was due to FRET quenching upon assembly of a H-bonded duplex between pDDDDDDDDDDDDf and pD*AAAAAAAAAAAAq, and to glean some information about the cooperativity associated with duplex formation in this system. Titration of DMSO into a 1 μM solution of a 1 : 1 mixture of pDDDDDDDDDDDDf and pD*AAAAAAAAAAAAq in chloroform (see Fig. S18†) led to an increase in the fluorescence intensity, due to denaturation of the duplex and a consequent increase in the physical distance between the dansyl fluorophore and dabcyl quencher.

The concentration of DMSO at the end of the denaturation experiment was nearly 1 M, so a control experiment was carried out using pure pDDDDDDDDDDDDf to confirm that high concentrations of DMSO do not significantly affect the properties of the fluorophore (see Fig. S18†). Small changes in the fluorescence intensity observed in the control experiment were used to correct the denaturation data: the fluorescence intensity measured for the duplex was subtracted from the fluorescence intensity measured for the control sample for each DMSO concentration to give Δ*I*. Fig. 7(b) shows the fluorescence intensity data from the denaturation experiment plotted as a function of DMSO concentration. Denaturation occurs as a sharp transition over a relatively narrow range of DMSO concentration. It was not possible to fit the data to a simple two-state, all-or-nothing denaturation isotherm, *i.e.* considering only the fully assembled duplex and the fully denatured complex, which indicates that some intermediate partially denatured species must be populated.

The degree of cooperativity associated with the denaturation transition can be quantified using a Hill plot (Fig. 7(c)). The fraction of pDDDDDDDDDDDDf bound to DMSO, *θ*, was determined from the data in Fig. 7(b) as (Δ*I*_max_ − Δ*I*)/Δ*I*_max_, where Δ*I*_max_ = 115, and the resulting Hill plot gives a Hill coefficient of 4.6 for DMSO denaturation of the pDDDDDDDDDDDDf·pD*AAAAAAAAAAAAq duplex (*i.e.* the slope at *θ* = 0.5). If each base-pair in the duplex were independent and there was no cooperativity, then the Hill coefficient would be 1. If denaturation was an all-or-nothing two-state process, then the Hill coefficient for the 12-mer duplex would be 12.^20^ The value of 4.6 therefore implies that there is significant cooperativity in assembly of the 12-mer duplex.

The degree of cooperativity associated with duplex formation can also be assessed from the switching window, which is the factorial increase in denaturant concentration required to change the duplex : single strand ratio from 10 : 1 to 1 : 10 (*c*_R_).^20^ For a non-cooperative system, the value of log *c*_R_ is 2, *i.e.* a 100-fold increase in DMSO concentration would be required to go from 90% duplex to 90% single strand. The value of log *c*_R_ determined from the data in Fig. 7 is 0.4, which indicates a highly cooperative process, where switching takes place over a 3-fold increase in DMSO concentration.

We have previously reported the DMSO denaturation of a 6-mer duplex in chloroform, and it is possible to use these data to quantify the cooperativity associated with formation of the shorter duplex (see Fig. S19†).^18^ The Hill coefficient is 1.9, and the switching window log *c*_R_ is 1.0, so the switch from duplex to single strand takes place over a 10-fold increase in DMSO concentration. These values indicate that assembly of the 6-mer duplex is also cooperative, but it is clear that there is a significant increase in the degree of cooperativity associated with assembly of the longer 12-mer duplex. The increase in the cooperativity of REMO duplex formation with increasing oligomer length parallels the observations for nucleic acid duplexes and suggests that truly all-or-nothing processes may emerge for longer REMO.^36^

## Conclusions

Synthetic polymers featuring an alternating 1,3,5-triazine–piperazine backbone and a sequence defined by side-chains carrying a phenol or phosphine oxide group were previously shown to form sequence-selective duplexes. Here, using several techniques, we demonstrate that longer REMO form duplexes in a highly cooperative manner. Automated SPS was used to synthesise homo-oligomers with the sequence zDDDDDDDDDDDDy and zD*AAAAAAAAAAAAy. ^31^P NMR spectroscopy showed that in a 1 : 1 mixture of these species, all the phosphine oxide side-chains were involved in H-bonding interactions. The zDDDDDDDDDDDDy·zD*AAAAAAAAAAAAy duplex was covalently trapped by equipping the ends of the oligomers with an azide and an alkyne group and using a CuAAC reaction. Even when very high concentrations of competing azide were used in the trapping experiment, significant duplex product persisted, showing that there is a high effective molarity for the intramolecular CuAAC reaction in the duplex.

FRET quenching experiments were exploited as a novel method for characterising duplex formation in chloroform. The two 12-mer homo-oligomers were appended with a fluorophore (f) and a quencher (q). A fluorescence titration of pD*AAAAAAAAAAAAq into pDDDDDDDDDDDDf led to a decrease in the intensity of the fluorescent signal, and the data fit well to a 1 : 1 binding isotherm giving an association constant of greater than 10^8^ M^−1^ in chloroform. A DMSO denaturation of a 1 : 1 mixture of the two complementary 12-mers showed that FRET quenching was indeed due to hybridisation of the oligomers into a H-bonded duplex. The Hill coefficient for the DMSO denaturation experiment was 4.6, which indicates that 12-mer duplex assembly is highly cooperative compared with assembly of a 6-mer duplex for which the Hill coefficient was only 1.9. In nature, cooperativity is a key organising principle to modulate molecular recognition and self-assembly in complex functional structures. As we attempt to design sequence-controlled synthetic polymers with functional properties, the cooperativity exhibited by the REMO architecture could be an important feature in helping to mimic the all-or-nothing behaviour seen in biological assemblies.

## Data availability

All supporting data is provided in the ESI.†

## Author contributions

The manuscript was written through contributions of all authors.

## Conflicts of interest

There are no conflicts to declare.

## Supplementary Material

SC-016-D4SC08591D-s001
