# Supplementary material for: Cooperativity in the assembly of H-bonded duplexes of synthetic recognition-encoded melamine oligomers
Source: Chem Sci. 2025 Mar 7;16(14):5995–6002. doi: 10.1039/d4sc08591d (PMC11886990; doi:10.1039/d4sc08591d)
Supplement: SC-016-D4SC08591D-s001 [file SC-016-D4SC08591D-s001.pdf]

Supplementary Information (SI) for Chemical Science.
This journal is © The Royal Society of Chemistry 2025

Cooperativity in the assembly of H-bonded duplexes of synthetic recognition-encoded melamine oligomers

Mohit Dhiman,[1] Luis Escobar,[1] Joseph T. Smith,[1] Christopher A. Hunter [1,*]

[1]Yusuf Hamied Department of Chemistry, University of Cambridge, Cambridge CB2 1EW, U.K.

*Email: herchelsmith.orgchem@ch.cam.ac.uk

Supplementary Information

1. **General Experimental Details**............S2
2. **Synthesis of Building Blocks**............S3
3. **Functionalisation of Wang Resin**............S10
4. **Protocols for Automated Solid-Phase Synthesis**............S11
5. **Synthesis of Oligomers**............S13
6. **CuAAC Duplex Trapping Experiments**............S23
7. **Fluorescence Experiments**............S25
8. **^{31}P NMR Experiments**............S29

1. General Experimental Details

All reagents and materials used in the syntheses described were purchased from commercial suppliers and used without prior purification. Dry solvents were obtained from a Grubbs PS-MD-5 solvent purification system and used with no further degassing. Thin layer chromatography (TLC) was carried out using silica gel 60F (Merck) on glass plates. LCMS analyses of samples were performed using a Waters Acquity H-class UPLC coupled with a single quadrupole Waters SQD2. Two different UPLC columns were used: an Acquity UPLC CSH C18 Column (130 Å, 1.7 µm, 2.1 mm x 50 mm), and an Acquity UPLC PRM PR BEH C4 Column (300 Å, 1.7 µm, 2.1 mm x 50 mm).

Purification of compounds by silica column chromatography was performed using an automated system (Combiflash® Rf+ or Combiflash® Rf+ Lumen) with pre-packaged silica cartridges (25 µm or 50 µm PuriFlash® columns). All NMR spectra were recorded using a Bruker 500 MHz Avance III Smart Probe Spectrometer, a Bruker 400 MHz Avance III HD Spectrometer, a Bruker 400 MHz Avance III HD Smart Probe Spectrometer, or a Bruker 400 MHz Neo Prodigy Spectrometer at 298 ± 0.1 K. The residual ^{1}H form of the solvent was used as the internal standard for referencing. In $CDCl_3$, the ^{1}H NMR spectra were referenced to δ 7.26 ppm and ^{13}C NMR spectra referenced to δ 77.16 ppm. In 1,1,2,2-tetrachloroethane-d_2, the ^{1}H NMR spectra were referenced to δ 6.00 ppm. In DMSO-d_6, the ^{1}H NMR spectra were referenced to δ 2.50 ppm. Chemical shifts (δ) are quoted in ppm and coupling constants (*J*) quoted in Hz. Splitting patterns are reported as: s (singlet), bs (broad singlet), d (doublet), t (triplet), q (quartet) and m (multiplet). FT-IR spectra were aquired with an ALPHA FT-IR Spectrometer from Bruker. HRMS spectra were recorded using a Waters SQD2 with Waters H-Class UPLC, equipped with a Waters Acquity UPLC BEH C18 Column (130 Å, 1.7µm, 2.1 mm x 50 mm). Analytical reverse-phase HPLC was performed on an Agilent HP-1100 Series HPLC system. Preparative reverse-phase HPLC was performed on an Agilent HP-1100 Series preparative HPLC system. UV-vis spectra were recorded on an Agilent Cary 60 UV-vis spectrophotometer controlled by Cary WinUV software.Fluorescence spectra were recorded using a Cary Eclipse Fluorescence Spectrophotometer G9800A (Agilent) controlled by Cary Eclipse software.

2. Synthesis of Building Blocks

Synthesis of 1

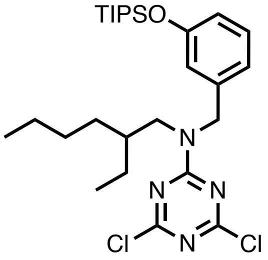

1

Compound **1** was synthesised according to the literature procedure found at:

M. Dhiman, R. Cabot and C. A. Hunter, *Chem. Sci.*, 2024, **15**, 5957–5963.

Synthesis of 2, 3 and 4

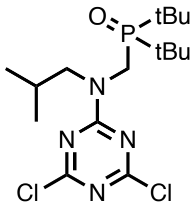

2

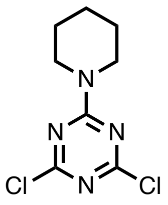

3

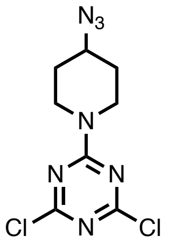

4

Compounds **2**, **3** and **4** were synthesised according to the literature procedures found at:

M. Dhiman, R. Cons, O. N. Evans, J. T. Smith, C. J. Anderson, R. Cabot, D. O. Soloviev, and C. A. Hunter, *J. Am. Chem. Soc.*, 2024, **146**, 9236–9334.

Synthesis of 5 and 6

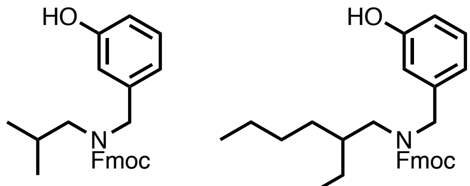

5 **6**

Compounds **5** and **6** were synthesised according to the literature procedures found at:

M. Dhiman, R. Cabot and C. A. Hunter, *Chem. Sci.*, 2024, **15**, 5957–5963.

Synthesis of f-N_3

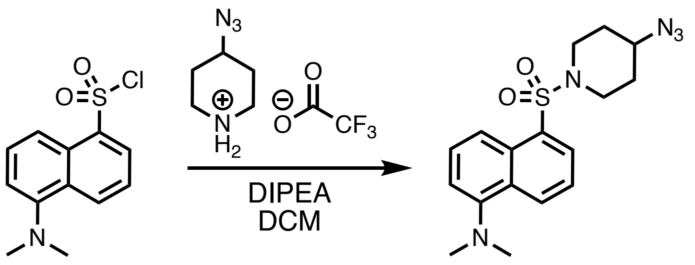

4-Ethynylpiperidine salt (0.30 g, 1.25 mmol, 1 equiv.) and dansyl chloride (0.67 g, 2.50 mmol, 2 equiv.) were dissolved in dry DCM (10 mL) under nitrogen atmosphere. DIPEA (0.45 mL, 2.50 mmol, 2 equiv.) was added. The reaction was stirred at r.t. overnight. After that, the crude mixture was diluted with DCM (10 mL) and washed with aq. sat. $NaHCO_3$ (20 mL). The crude was further extracted with DCM (2 × 10 mL). The combined organic layers were dried ($MgSO_4$), filtered and concentrated. The crude product was purified by flash chromatography (SiO_2, 50-100% gradient of DCM in 40-60 petroleum ether) to yield the pure product **f-N_3** (0.18 g, 0.50 mmol, 40%) as a green oil.

^{1}H NMR (400 MHz, chloroform-*d*): δ_H 8.57 (d, J = 8.5 Hz, 1H), 8.35 (d, J = 8.5 Hz, 1H), 8.21 (dd, J = 7.4 Hz, J = 1.3 Hz, 1H), 7.56-7.51 (m, 2H), 7.19 (d, J = 7.4 Hz, 1H), 3.55-3.48 (m, 3H), 3.10-3.04 (m, 2H), 2.89 (s, 6H), 1.95-1.88 (m, 2H), 1.70-1.62 (m, 2H);
^{13}C NMR (101 MHz, chloroform-*d*): δ_C 151.9, 133.3, 130.9, 130.7, 130.4, 130.2, 128.2, 123.3, 119.7, 115.4, 56.6, 45.6, 43.1, 30.3;
HRMS (ES+): calculated for $C_{17}H_{21}N_5O_2S$ 360.1488 $[M+H]^+$, found 360.1475 $[M+H]^+$;
FT-IR (ATR): ν_{max}/cm^{-1} 2941, 2097, 1587, 1573, 1454, 1406, 1309, 1244, 1231, 1201, 1161, 1144, 1092, 1072.

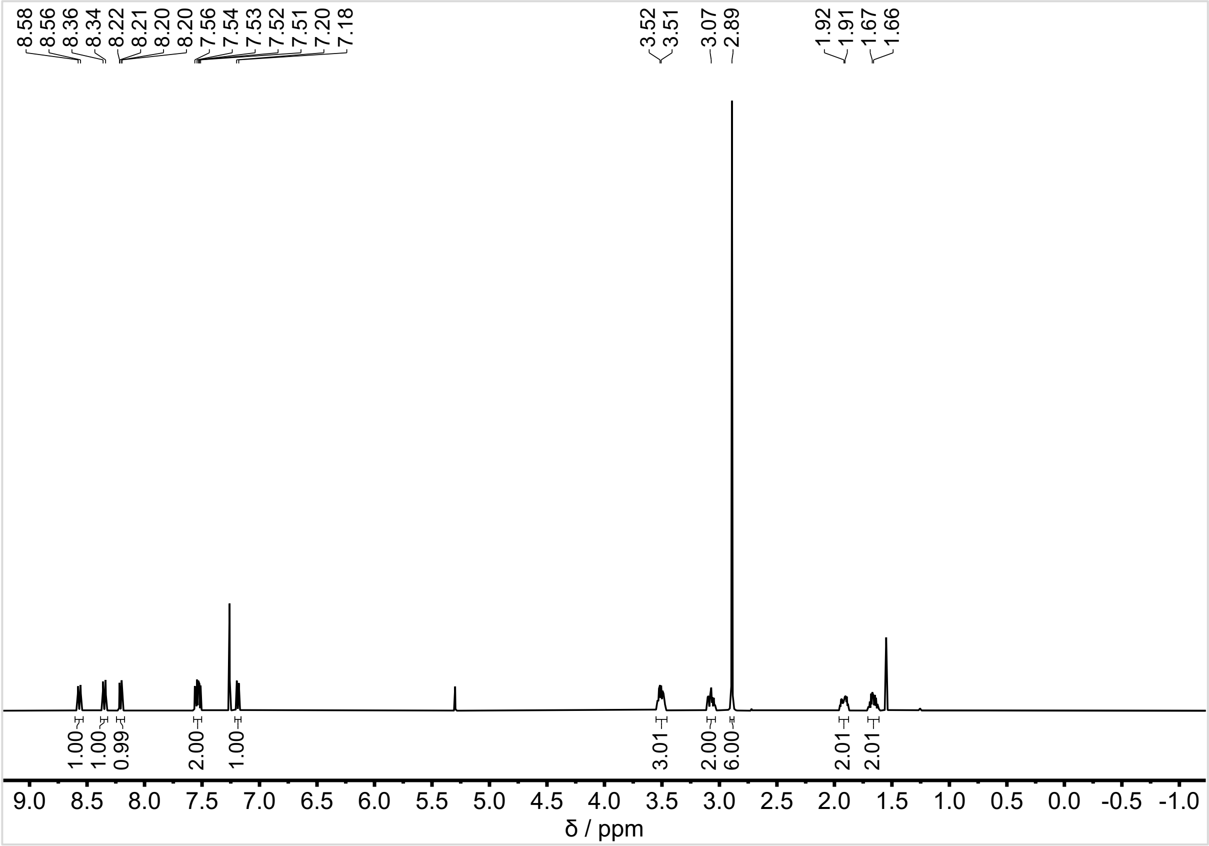

Figure S1: ^{1}H NMR (400 MHz, chloroform-*d*) spectrum of **f-N$_3$**.

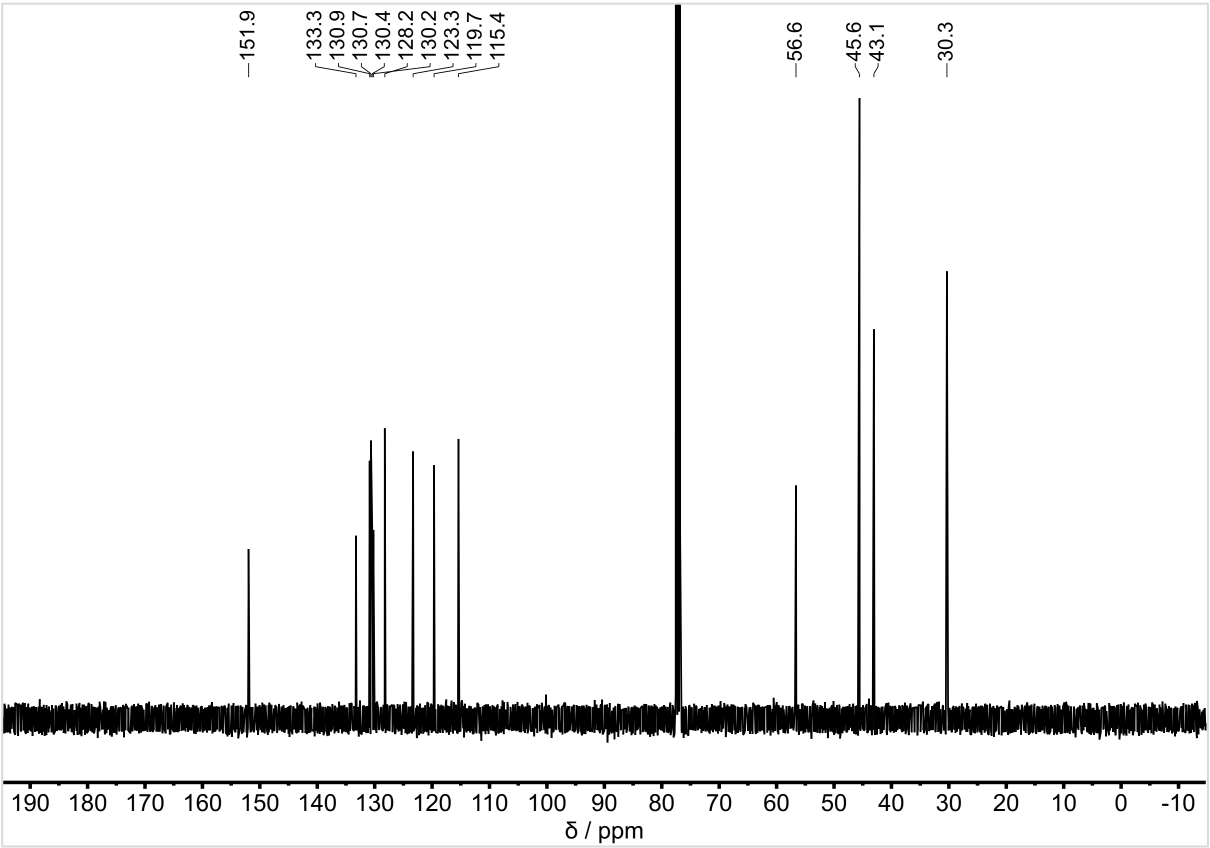

Figure S2: ^{13}C NMR (101 MHz, chloroform-*d*) spectrum of **f-N$_3$**.

Synthesis of q-N_3

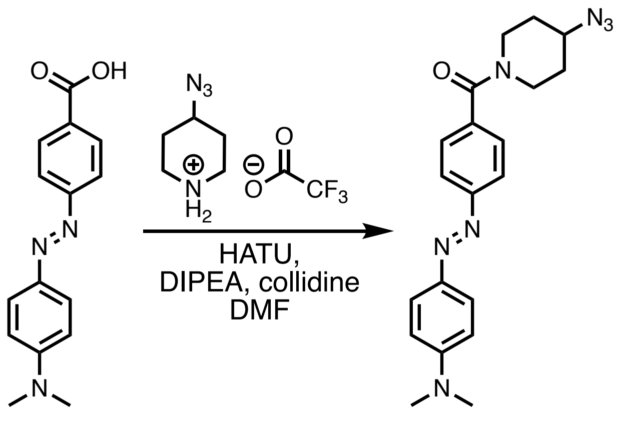

Dabcyl acid (0.10 g, 0.37 mmol, 1 equiv.), 4-ethynylpiperidine salt (0.36 g, 1.48 mmol, 4 equiv.) and HATU (0.56 g, 1.48 mmol, 4 equiv.) were dissolved in dry DMF (8 mL) under nitrogen atmosphere. Collidine (0.20 mL, 1.48 mmol, 4 equiv.) and DIPEA (0.51 mL, 2.96 mmol, 8 equiv.) were added. The reaction was stirred at r.t. for 1 h. After that, the reaction was stopped and the crude mixture was washed with aq. sat. $NaHCO_3$ (80 mL). The crude was further extracted with DCM (3 × 20 mL). The combined organic layers were dried ($MgSO_4$), filtered and concentrated. The crude product was purified by flash chromatography (SiO_2, 0-80% gradient of EtOAc in DCM) to yield the pure product **q-N_3** (0.14 g, 0.37 mmol, 98%) as a red solid.

^{1}H NMR (400 MHz, chloroform-*d*): δ_H 7.99 (d, J = 9.2 Hz, 2H), 7.85 (d, J = 8.5 Hz, 2H), 7.50 (d, J = 8.5 Hz, 2H), 6.76 (d, J = 9.2 Hz, 2H), 3.76-3.70 (m, 1H), 3.10 (s, 6H) (some proton signals of the piperidine group appeared too broad for an unequivocal assignment);
^{13}C NMR (101 MHz, chloroform-*d*): δ_C 170.3, 154.1, 152.9, 143.7, 136.1, 127.9, 125.5, 122.4, 111.6, 57.5, 40.4 (some carbon signals of the piperidine group appeared too broad for an unequivocal assignment);
HRMS (ES+): calculated for $C_{20}H_{24}N_7O$ 378.2037 $[M+H]^+$, found 378.2039 $[M+H]^+$;
FT-IR (ATR): ν_{max}/cm^{-1} 2923, 2096, 1631, 1600, 1519, 1498, 1438, 1422, 1393, 1364, 1312, 1276, 1243, 1138.

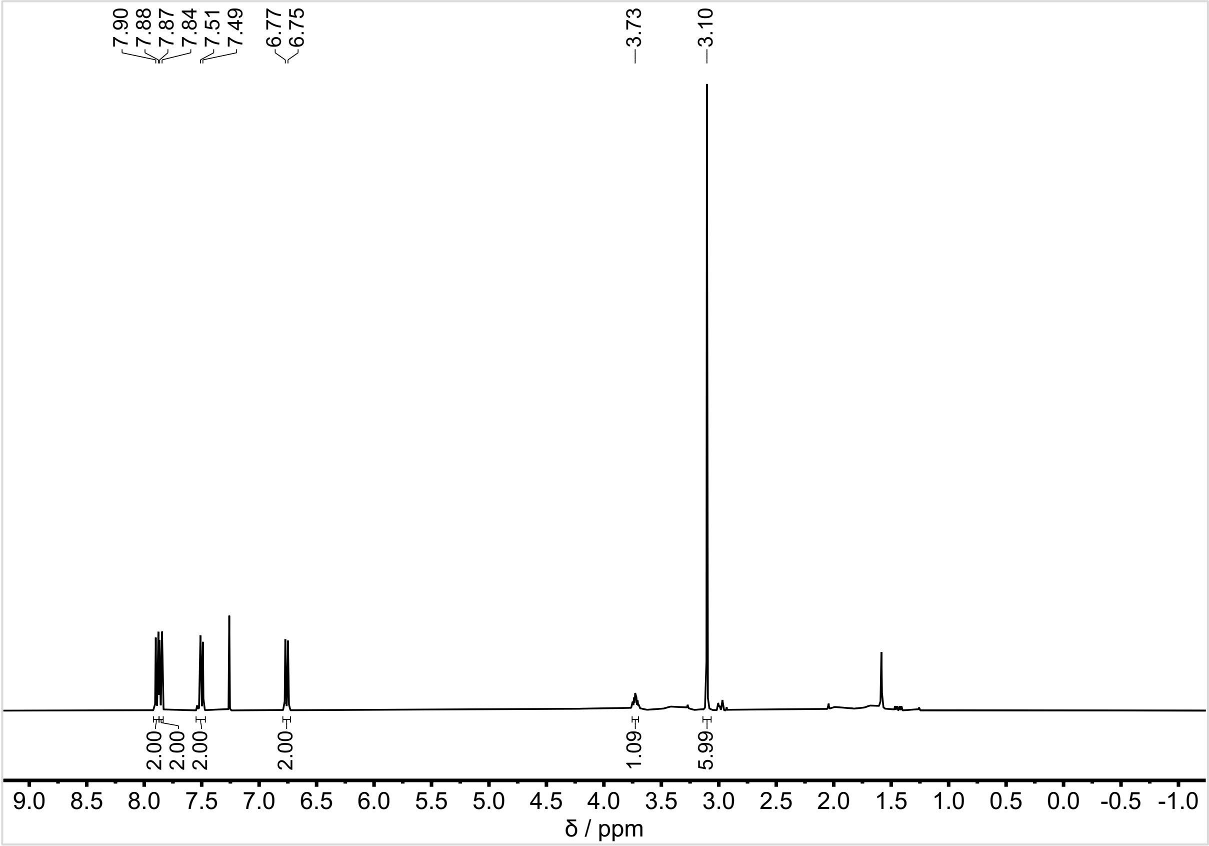

Figure S3: ^{1}H NMR (400 MHz, chloroform-*d*) spectrum of **q-N$_3$**.

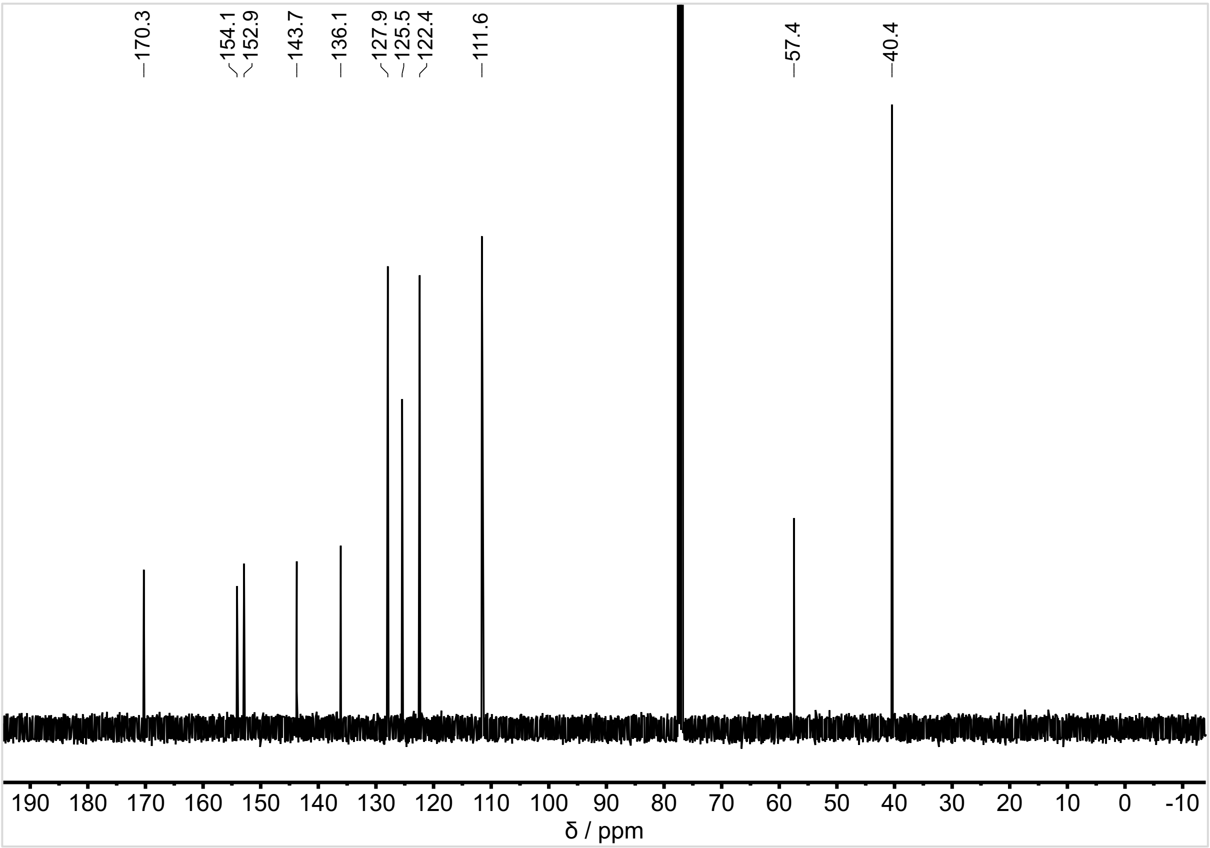

Figure S4: ^{13}C NMR (101 MHz, chloroform-*d*) spectrum of **q-N$_3$**.

Synthesis of pAp

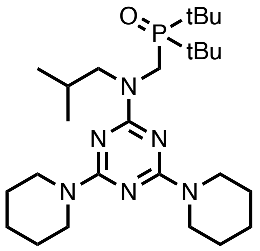

pAp was synthesised according to the literature procedure found at:

P. Troselj, P. Bolgar, P. Ballester, and C. A. Hunter, *J. Am. Chem. Soc.*, 2021*,* **143***,* 8669–8678.

3. Functionalisation of Wang Resin

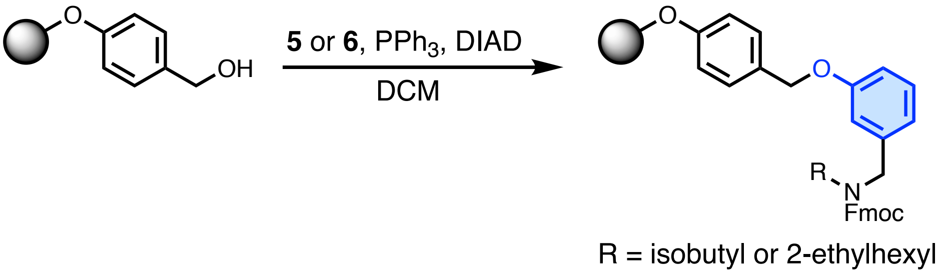

TentaGel Wang Resin (90 µm mesh) was swollen in dry DCM for 30 min before a solution of **5** or **6** and triphenylphosphine in dry DCM was added to the resin. A solution of diisopropyl azodicarboxylate was diluted 5-fold in dry DCM then added dropwise to the resin. The resin was agitated at r.t. overnight, and then washed alternatingly with DCM (5x) and DMF (5x) to yield the functionalised Wang resin.

Quantification of resin loading: Functionalised Wang resin was treated with a solution of DBU in DMF (2 mL, 2 vol. %) and agitated for 30 mins. 1 mL of the solution was removed from the resin and diluted with 4 mL of acetonitrile. A 1 mL aliquot of the resultant solution was taken and diluted to 12.5 mL with acetonitrile. The absorbance of the DBU-fulvene adduct (λ = 304 nm, ε = 9254 M^{-1} cm^{-1}) was measured to estimate the resin loading.

4. Protocols for Automated Solid-Phase Synthesis

General methods using CEM Liberty Blue Automated Synthesiser: Heated automated Solid-Phase Synthesis (SPS) was performed on a CEM Liberty Blue automated synthesiser on a 50 µmol scale. DMF solutions of piperazine (0.7 M), **1** (0.125 M), **2** (0.125 M), **3** (0.125 M), **4** (0.125 M) and DIPEA (0.5 M) were prepared for coupling. General synthetic protocols performed were:

Fmoc deprotection: The loaded Wang resin was agitated in a solution of piperazine in DMF (7 mL, 0.7 M, 2 x 10 min). The deprotection solution was then drained and the resin was washed with DMF (4 × 5 mL).

Coupling cycle: The resin-bound oligomer was first agitated in a solution of **1**, **2**, **3** or **4** (0.1 M, 10 eq.) and DIPEA (0.1 M, 10 eq.) in DMF (5 mL) for 10 or 15 mins at 90 °C. The 1st coupling solution was drained, and the resin was washed with DMF (4 × 5 mL). The resin-bound oligomer was then agitated in a solution of piperazine (5 mL, 0.7 M) in DMF for 10 or 15 min at 90 °C. The 2nd coupling solution was drained, and the resin was washed with DMF (4 × 5 mL).

4-Ethynylpiperidine coupling (manual MW-assisted SPS): The resin-bound oligomer was swollen in DMF for 15 min before being agitated in a solution of 4-ethynylpiperidine trifluoroacetate (0.1 M) and DIPEA (1 M) in DMF (5 mL) for 30 mins at 90 °C. The coupling solution was drained, and the resin was washed with DMF (4 × 5 mL).

TIPS deprotection: The resin-bound oligomer was swollen in THF for 15 min before being agitated in a solution of TBAF (5 mL, 1M in THF) at r.t. for 1h. The solution was drained, and the resin was washed with MeOH and THF alternatingly (4 × 5 mL) before being subjected to another deprotection cycle.

Resin Cleavage: The resin was agitated in a mixture of TFA:TIS:DCM (90:5:5 v/v/v) at r.t. for 2 h. The resin was filtered and washed with DCM (5 × 5 mL) and then subjected to another cleavage cycle. The combined filtrates from both cleavage cycles were concentrated under N_2 flow before drying *in vacuo*.

HPLC Purification: Analytical reverse-phase HPLC was performed on an Agilent HP-1100 Series HPLC system composed of a high-pressure binary pump, an autosampler with injector programming capabilities and a diode array detector with a semimicro flow cell (6 mm path length, 5 µL volume). UV/vis absorption was measured at 240 nm and 280 nm (8 nm bandwidth). A Waters XBridge BEH C8 (130 Å, 2.5 µm), 6 x 75 mm column was employed at a flow rate of 0.7 or 0.8 mL/min.

Preparative reverse-phase HPLC was performed on an Agilent HP-1100 Series preparative HPLC system composed of a high-pressure mixing binary pump, dual injector autosampler loops (50 µL/5 mL loops), a variable UV/vis detector (190 nm to 600 nm) and an automatic fraction collector. UV/vis absorption was measured at 240 nm and 280 nm (8 nm bandwidth) and fractions were set to collect automatically based on retention time. A Waters XBridge BEH C8 OBD Prep (130 Å, 5 µm), 19 x 250 mm column was employed at a flow rate of 10 or 12 mL/min.

Both instruments utilised a mobile phase composed of 95:5 water (HPLC grade):MeCN (HPLC grade) as solvent A, and THF (HPLC grade) as solvent B operating using a linear gradient.

5. Synthesis of Oligomers

Synthesis of zDDDDDDDDDDDDy and zDAAAAAAAAAAAAy

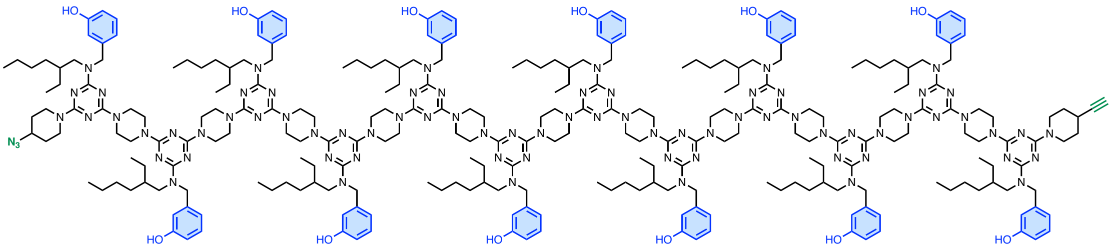

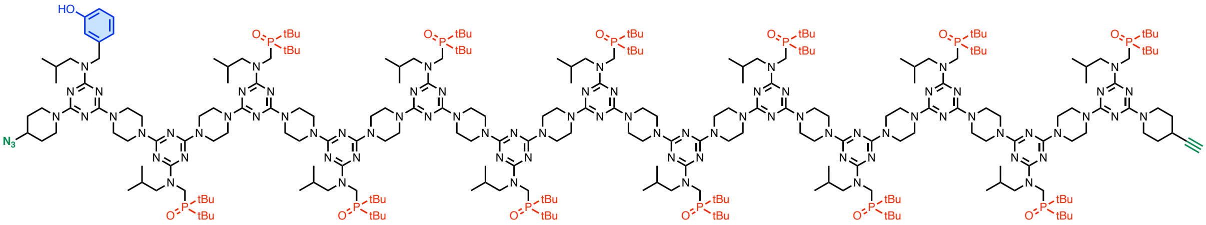

zDDDDDDDDDDDDy and **zDAAAAAAAAAAAAy** were synthesised according to the literature procedures found at:

M. Dhiman, R. Cabot and C. A. Hunter, *Chem. Sci.*, 2024, **15**, 5957–5963.

Synthesis of zD*AAAAAAAAAAAAy

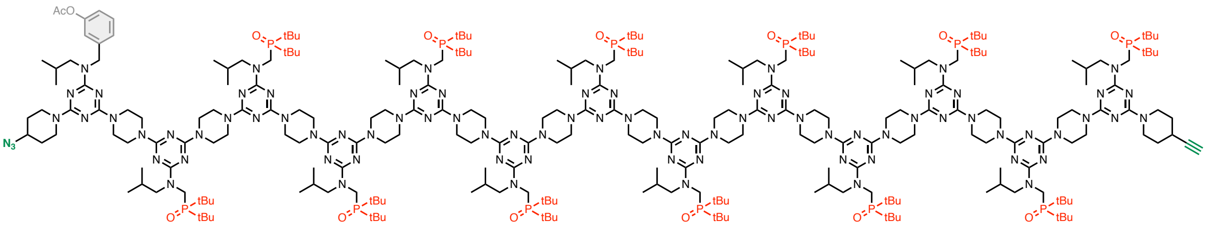

A mixture of **zDAAAAAAAAAAAAy** (30 mg, 0.006 mmol), glacial acetic acid (3 mg, 0.056 mmol), EDC•HCl (11 mg, 0.056 mmol) and DMAP (7 mg, 0.056 mmol) in dry THF (4 mL) was stirred at r.t. overnight. The mixture was extracted with EtOAc (3x) and washed with 1 M HCl (2x), water and brine. The organic phase was dried over $MgSO_4$ and the solvent was removed *in vacuo* to yield the crude product. The crude was purified by flash chromatography (SiO_2, 0-20% gradient of methanol in DCM) to yield the pure product **zD*AAAAAAAAAAAAy** (29 mg, 0.006 mmol, 95%) as a white solid.

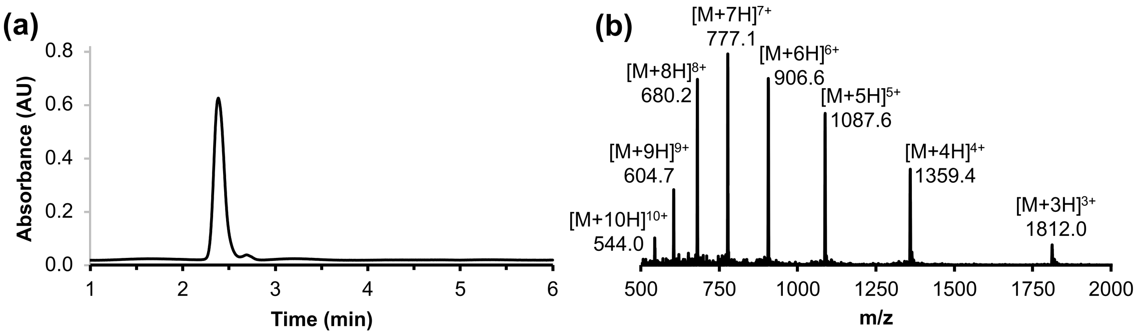

Figure S5: (a) UPLC trace of **zD*AAAAAAAAAAAAy**. **(b)** ESI-MS **zD*AAAAAAAAAAAAy**. Calculated mass: 1812.2 $[M+3H]^{3+}$, 1359.4 $[M+4H]^{4+}$, 1087.7 $[M+5H]^{5+}$, 906.6 $[M+6H]^{6+}$, 771.2 $[M+7H]^{7+}$, 680.2 $[M+8H]^{8+}$, 604.7 $[M+9H]^{9+}$, 544.4 $[M+10H]^{10+}$. *UPLC Conditions:* C4 column at 40 °C using a 30-100% gradient of THF/formic acid (0.1%) in water/formic acid (0.1%) over 4 minutes, then 100% THF/formic acid (0.1%) over 2 minutes.

^{1}H NMR (400 MHz, chloroform-*d*): δ_H 7.31-6.69 (aryl CH, 4H) 4.86-3.08 (-NC<u>H</u>$_2$-, 157H), 2.66 ((HC≡C)C<u>H</u>, 1H), 2.25-1.55 (-C<u>H</u>$_2$, *i*-Bu -C<u>H</u> & -C≡C<u>H</u>, 26H), 1.34-1.23 (*t*-butyl C<u>H</u>/C<u>H</u>$_3$, 216H) 0.93-0.86 (*i*-Bu -C<u>H</u>$_3$, 78H);

^{31}P NMR (162 MHz, chloroform-*d*): δ_P 58.87;

HRMS (ES+): calculated for $C_{268}H_{481}N_{81}O_{14}P_{12}$ 2717.8249 $[M+2H]^{2+}$, found 2717.8200 $[M+2H]^{2+}$.

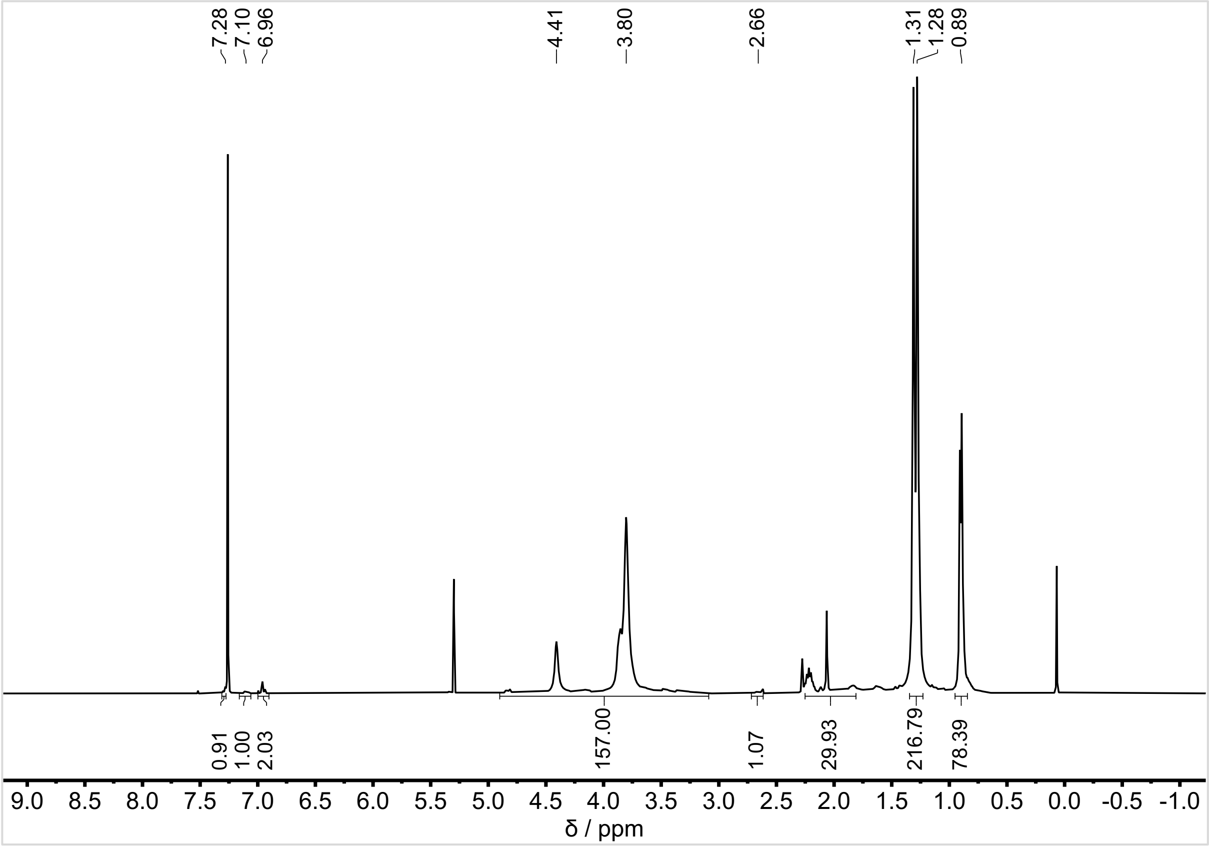

Figure S6: ^{1}H NMR (400 MHz, chloroform-*d*) spectrum of **zD*AAAAAAAAAAAAy**.

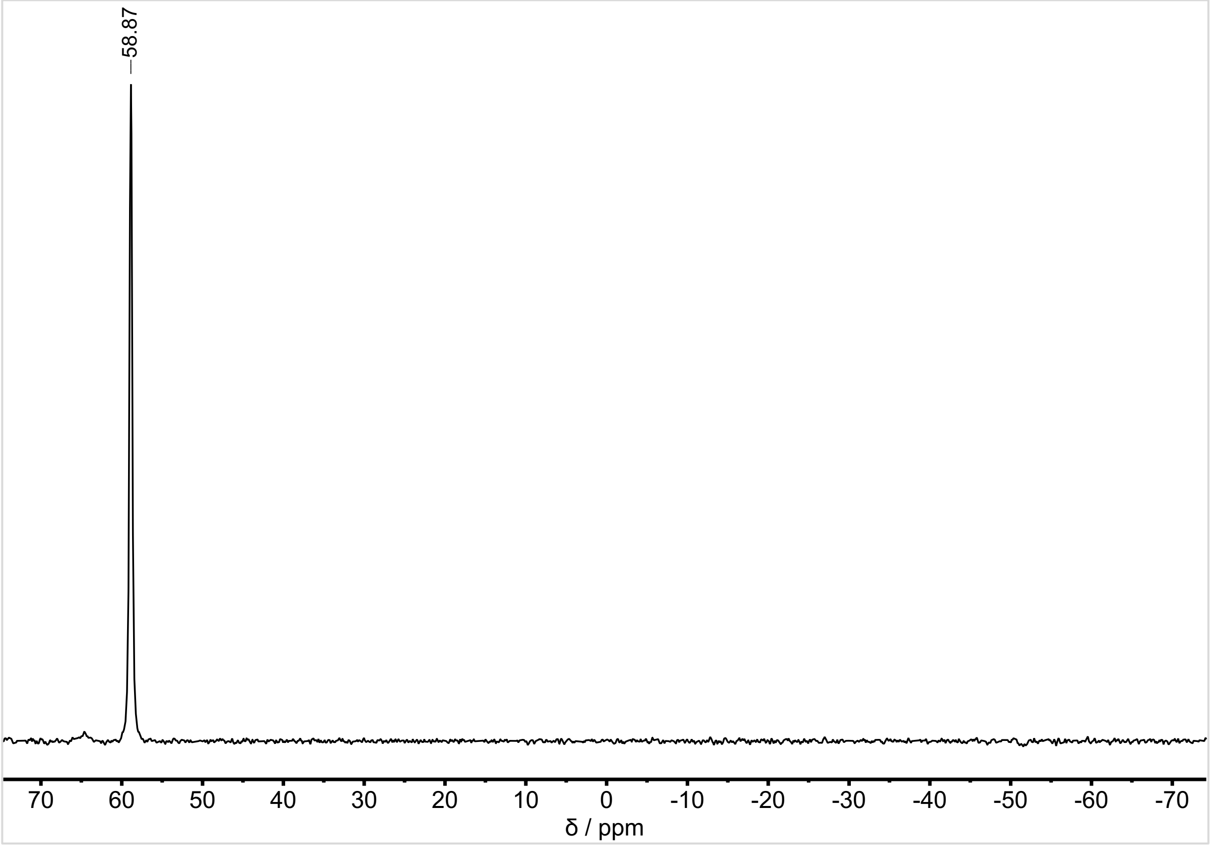

Figure S7: ^{31}P NMR (162 MHz, chloroform-*d*) spectrum of **zD*AAAAAAAAAAAAy**.

Synthesis of pDDDDDDDDDDDDy

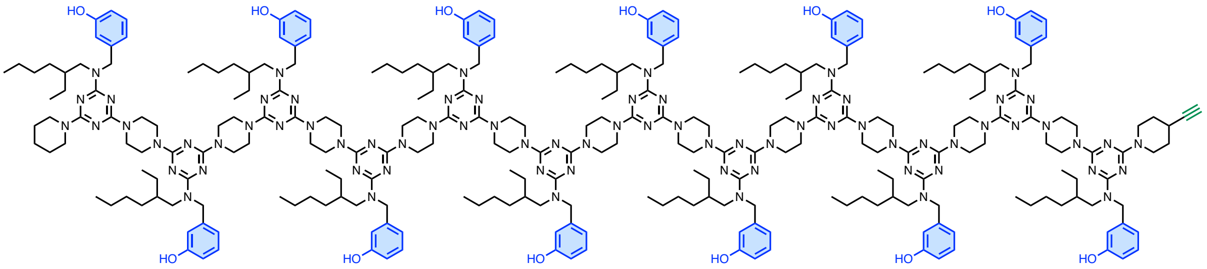

TentaGel Wang resin was loaded with **6** in accordance with the general methods. The loaded resin (50 µmol) was subjected to SPS on a CEM Liberty Blue automated synthesiser, followed by coupling with 4-ethynylpiperidine via manual SPS. TIPS deprotection followed by resin cleavage afforded the crude oligomer. The crude oligomer was purified via preparative HPLC (72.5% B over 50 mins) to yield the oligomer **pDDDDDDDDDDDDy** (41 mg, 17% based on the initial resin loading) as a white solid.

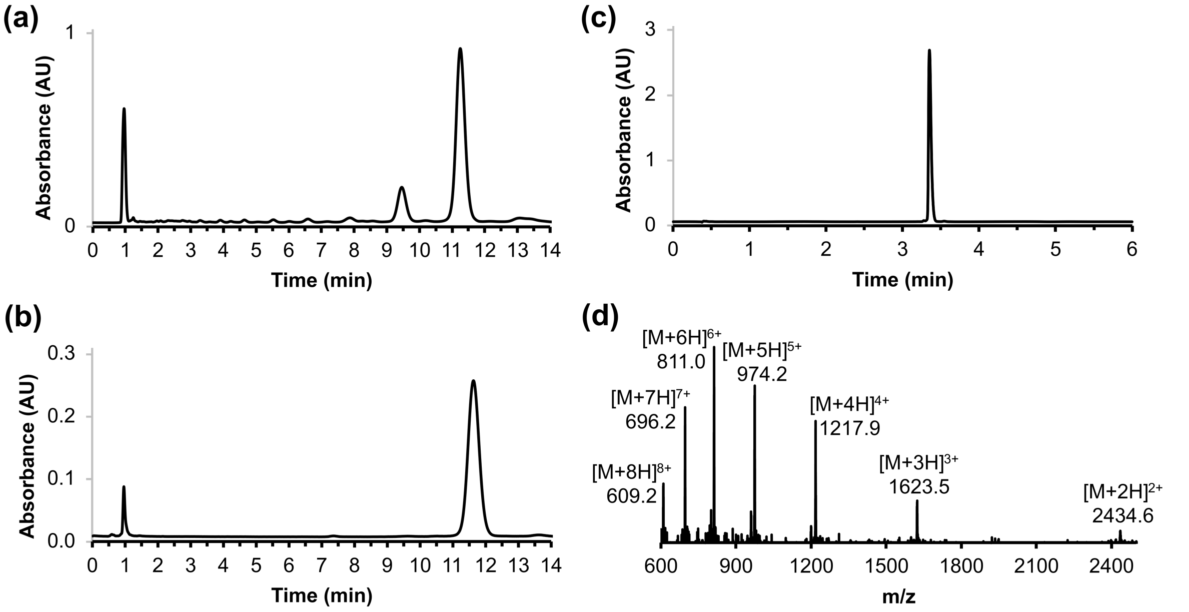

Figure S8: (a) Crude analytical HPLC trace of **pDDDDDDDDDDDDy**. **(b)** Analytical HPLC trace of **pDDDDDDDDDDDDy** after purification. **(c)** UPLC trace of **pDDDDDDDDDDDDy** after purification. **(d)** ESI-MS of **pDDDDDDDDDDDDy**. Calculated mass: 2434.6 $[M+2H]^{2+}$, 1623.4 $[M+3H]^{3+}$, 1217.8 $[M+4H]^{4+}$, 974.3 $[M+5H]^{5+}$, 811.0 $[M+6H]^{6+}$, 696.2 $[M+7H]^{7+}$, 609.2 $[M+8H]^{8+}$. *UPLC Conditions:* C4 column at 40 °C using a 30-100% gradient of THF/formic acid (0.1%) in water/formic acid (0.1%) over 4 minutes, then 100% THF/formic acid (0.1%) over 2 minutes.

^{1}H NMR (400 MHz, DMSO-*d₆*): δ_H 8.73 (br s, 12H), 7.05 (t, J = 8.6 Hz, 12H), 6.68 (s, 12H), 6.69-6.61 (m, 24H), 4.75 (d, J = 15.4 Hz, 12H), 4.68 (d, J = 15.4 Hz, 12H), 3.75-2.90(-NC<u>H</u>₂- region), 1.86-1.74 (m, 12H), 1.43-1.18 (-C<u>H</u>₂- region), 0.92-0.80 (m, -C<u>H</u>₃ region 72H); **HRMS (ES+):** calculated for $C_{272}H_{396}N_{72}O_{12}$ 2434.1416 $[M+2H]^{2+}$, found 2434.1414 $[M+2H]^{2+}$.

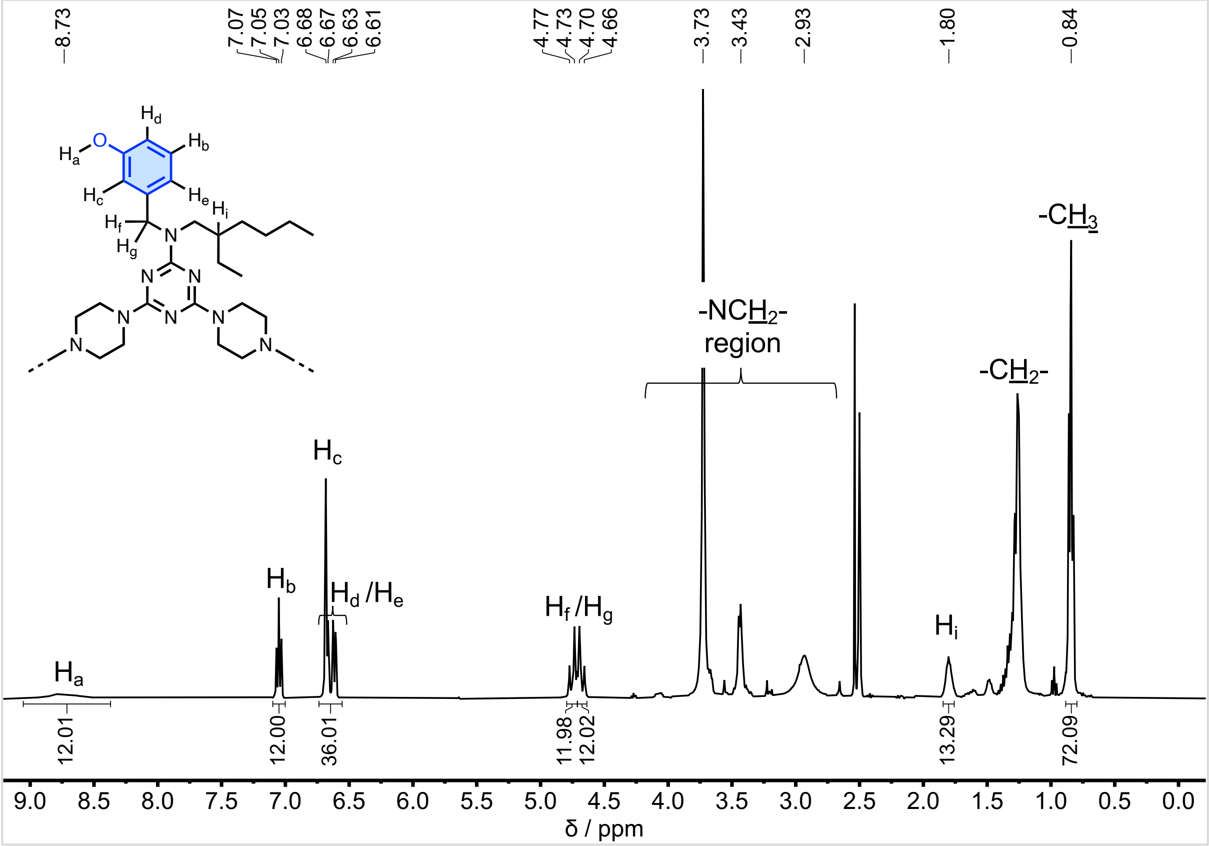

Figure S9: ^{1}H NMR (400 MHz, DMSO-d_6, 373 K) spectrum of **pDDDDDDDDDDDDy**.

Synthesis of pDDDDDDDDDDDDf

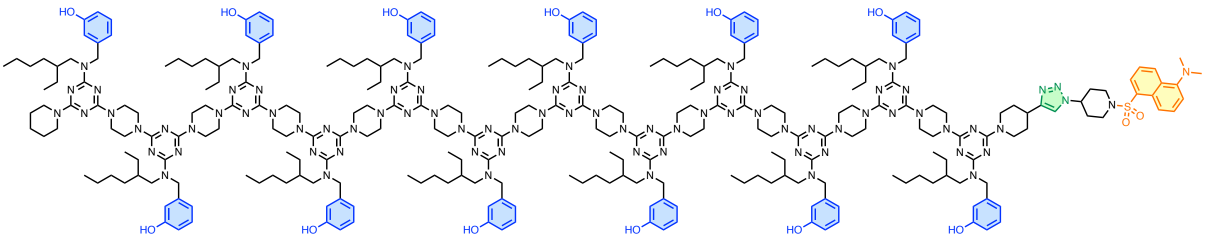

A mixture of **pDDDDDDDDDDDDy** (15 mg, 0.003 mmol), **f-N$_3$** (12 mg, 0.031 mmol), $Cu(MeCN)_4PF_6$ (1 mg, 0.002 mmol) and sodium ascorbate (1 mg, 0.003 mmol) was dissolved in dry DMF (5 mL) under nitrogen atmosphere. The mixture was heated in a sealed vial in a microwave reactor for 10 min at 90 °C. The mixture was diluted with water then extracted with EtOAc (3×). The combined organic layers were washed with 1 M HCl (1×), 0.01 M EDTA (1×), 5% LiCl soln. (3×) and brine (1×). The organic phase was dried over $MgSO_4$ and then the solvent was removed *in vacuo* to obtain the crude product. The crude oligomer was purified via preparative HPLC (71.5% B over 40 mins) to yield the oligomer **pDDDDDDDDDDDDf** (7 mg, 0.001 mmol, 43%) as an off-white solid.

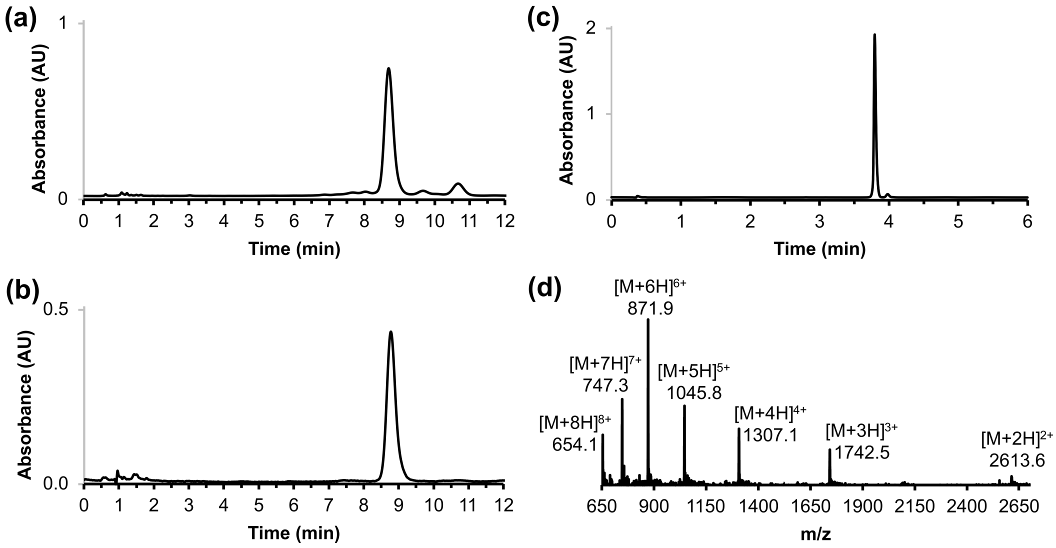

Figure S10: (a) Crude analytical HPLC trace of **pDDDDDDDDDDDDf**. **(b)** Analytical HPLC trace of **pDDDDDDDDDDDDf** after purification. **(c)** UPLC trace of **pDDDDDDDDDDDDf** after purification. **(d)** ESI-MS of **pDDDDDDDDDDDDf**. Calculated mass: 2613.7 $[M+2H]^{2+}$, 1742.5 $[M+3H]^{3+}$, 1307.1 $[M+4H]^{4+}$, 1045.9 $[M+5H]^{5+}$, 871.9 $[M+6H]^{6+}$, 747.3 $[M+7H]^{7+}$, 654.2 $[M+8H]^{8+}$. *UPLC Conditions:* C4 column at 40 °C using a 30-100% gradient of THF/formic acid (0.1%) in water/formic acid (0.1%) over 4 minutes, then 100% THF/formic acid (0.1%) over 2 minutes.

^{1}H NMR (400 MHz, DMSO-d_6): δ_H 8.77 (br s, 12H), 8.55 (d, J = 8.6 Hz, 1H), 8.34 (d, J = 8.7 Hz, 1H), 8.16 (dd, J = 7.3, 1.3 Hz, 1H), 7.70 (s, 1H), 7.67-7.57 (m, 2H), 7.27 (d, J = 7.5 Hz, 1H), 7.04 (t, J = 7.9 Hz, 12H), 6.67 (s, 12H), 6.67-6.60 (m, 24H), 4.75 (d, J = 15.3 Hz, 12H), 4.67 (d, J = 15.3 Hz, 12H), 3.95-2.75 (-NC<u>H</u>$_2$- region), 1.84-1.74 (m, 12H), 1.41-1.17 (m, -C<u>H</u>$_2$- region), 0.92-0.79 (m, 72H);

HRMS (ES+): calculated for $C_{290}H_{417}N_{77}O_{14}S$ 2613.7185 $[M+2H]^{2+}$, found 2613.7192 $[M+2H]^{2+}$.

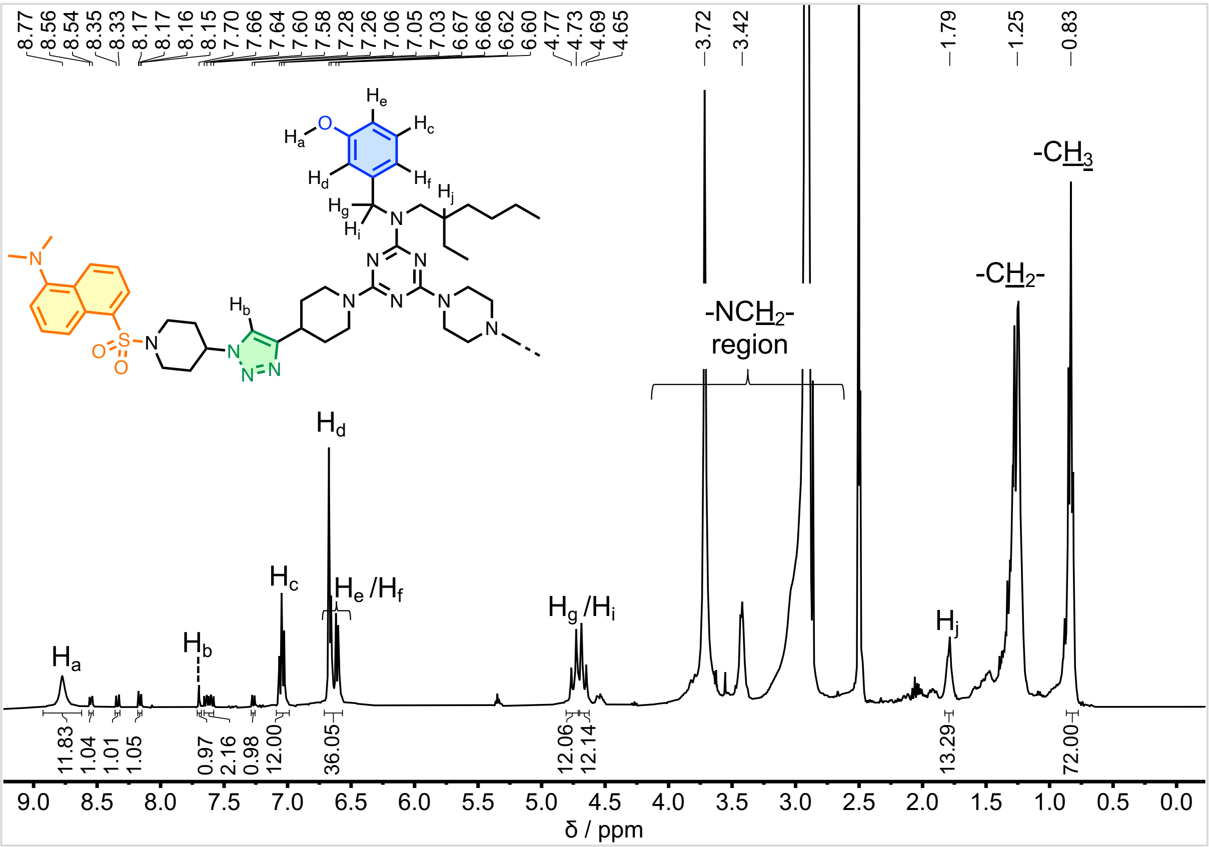

Figure S11: ^{1}H NMR (400 MHz, DMSO-d_6, 373 K) spectrum of **pDDDDDDDDDDDDf**.

Synthesis of pD*AAAAAAAAAAAAq

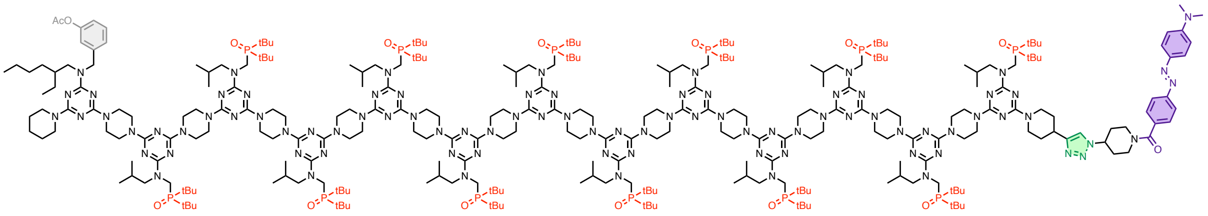

TentaGel Wang resin was loaded with **6** in accordance with the general methods. The loaded resin (36 µmol) was subjected to SPS on a CEM Liberty Blue automated synthesiser, followed by coupling with 4-ethynylpiperidine via manual SPS.

A mixture of the crude resin (0.036 mmol based on initial resin loading), **q-N$_3$** (27 mg, 0.072 mmol), $Cu(MeCN)_4PF_6$ (3 mg, 0.007 mmol) and sodium ascorbate (2 mg, 0.01 mmol) in dry DMF (5 mL) was prepared under nitrogen atmosphere. The mixture was heated in a sealed vial in a microwave reactor for 10 min at 90 °C. The coupling solution was drained, and the resin washed alternatingly with DMF and DCM (4 × 5 mL). The resin was cleaved in accordance with the general methods, and the collected filtrate was used in the next step without purification.

A mixture of the crude product from the previous step (0.036 mmol based on initial resin loading), glacial acetic acid (22 mg, 0.36 mmol), EDC•HCl (69 mg, 0.36 mmol) and DMAP (44 mg, 0.36 mmol) in dry THF (10 mL) was stirred at r.t. overnight. The mixture was extracted with EtOAc (3×) and washed with 1 M HCl (2×), water and brine. The organic phase was dried over $MgSO_4$ and the solvent was removed *in vacuo* to yield the crude product. The crude oligomer was purified via preparative HPLC (45-50% B over 40 mins) to yield the oligomer **pD*AAAAAAAAAAAAq** (40 mg, 0.007 mmol, 19%) as a red solid.

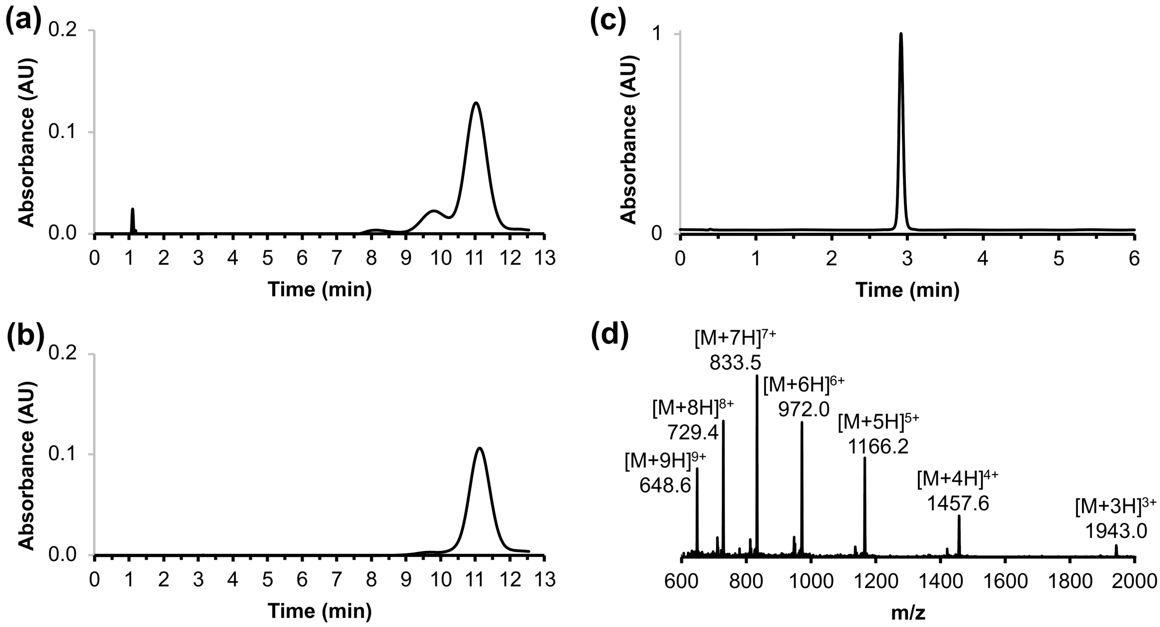

Figure S12: (a) Crude analytical HPLC trace of **pD*AAAAAAAAAAAAq**. **(b)** Analytical HPLC trace of **pD*AAAAAAAAAAAAq** after purification. **(c)** UPLC trace of **pD*AAAAAAAAAAAAq** after purification. **(d)** ESI-MS of **pD*AAAAAAAAAAAAq**. Calculated mass: 1943.0 $[M+3H]^{3+}$, 1457.6 $[M+4H]^{4+}$, 1166.2 $[M+5H]^{5+}$, 972.0 $[M+6H]^{6+}$, 833.4 $[M+7H]^{7+}$, 729.4 $[M+8H]^{8+}$, 648.5 $[M+9H]^{9+}$. *UPLC Conditions:* C4 column at 40 °C using a 30-100% gradient of THF/formic acid (0.1%) in water/formic acid (0.1%) over 4 minutes, then 100% THF/formic acid (0.1%) over 2 minutes.

^{1}H NMR (400 MHz, 1,1,2,2-tetrachloroethane-*d*$_2$): δ_H 7.93 (d, J = 9.3 Hz, 2H), 7.90 (d, J = 9.3 Hz, 2H), 7.57 (d, J = 8.2 Hz, 2H), 7.33 (s, 1H), 7.33-7.29 (m, 1H), 7.22-7.15 (m, 1H), 7.10 (s, 1H), 7.04-6.98 (m, 1H), 6.82 (d, J = 8.9 Hz, 2H), 4.88-4.85 (-N$C\underline{H}_2C_6H_5OH$, 2H), 4.46 (-N$C\underline{H}_2$PO(*t*-Bu)$_2$, 24H), 4.11-3.10 (piperazine -N$C\underline{H}_2$- region), 3.13 (s, 6H), 2.36-1.40 (*i*-Bu -C$\underline{H}$ & 2-ethylhexyl -C$\underline{H}_2$-), 1.37-1.32 (*t*-butyl C$\underline{H}$/C$\underline{H}_3$), 1.02-0.89 (-C$\underline{H}_3$);

^{31}P NMR (162 MHz, 1,1,2,2-tetrachloroethane-*d*$_2$): δ_P 58.52;

HRMS (ES+): calculated for $C_{292}H_{513}N_{85}O_{15}P_{12}$ 1942.9722 $[M+3H]^{3+}$, found 1942.9689 $[M+3H]^{3+}$.

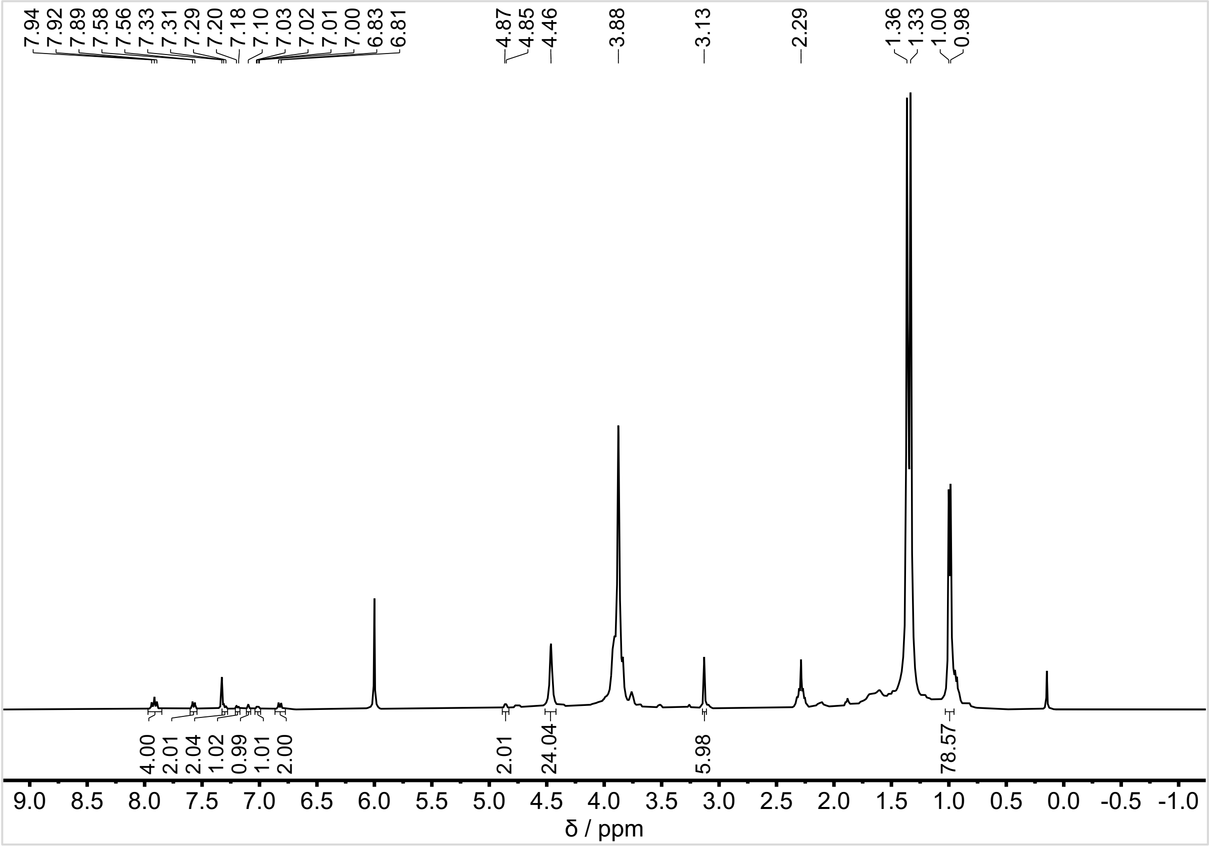

Figure S13: ^{1}H NMR (400 MHz, 1,1,2,2-TCE-d_2, 373 K) spectrum of **pD*AAAAAAAAAAAAq**.

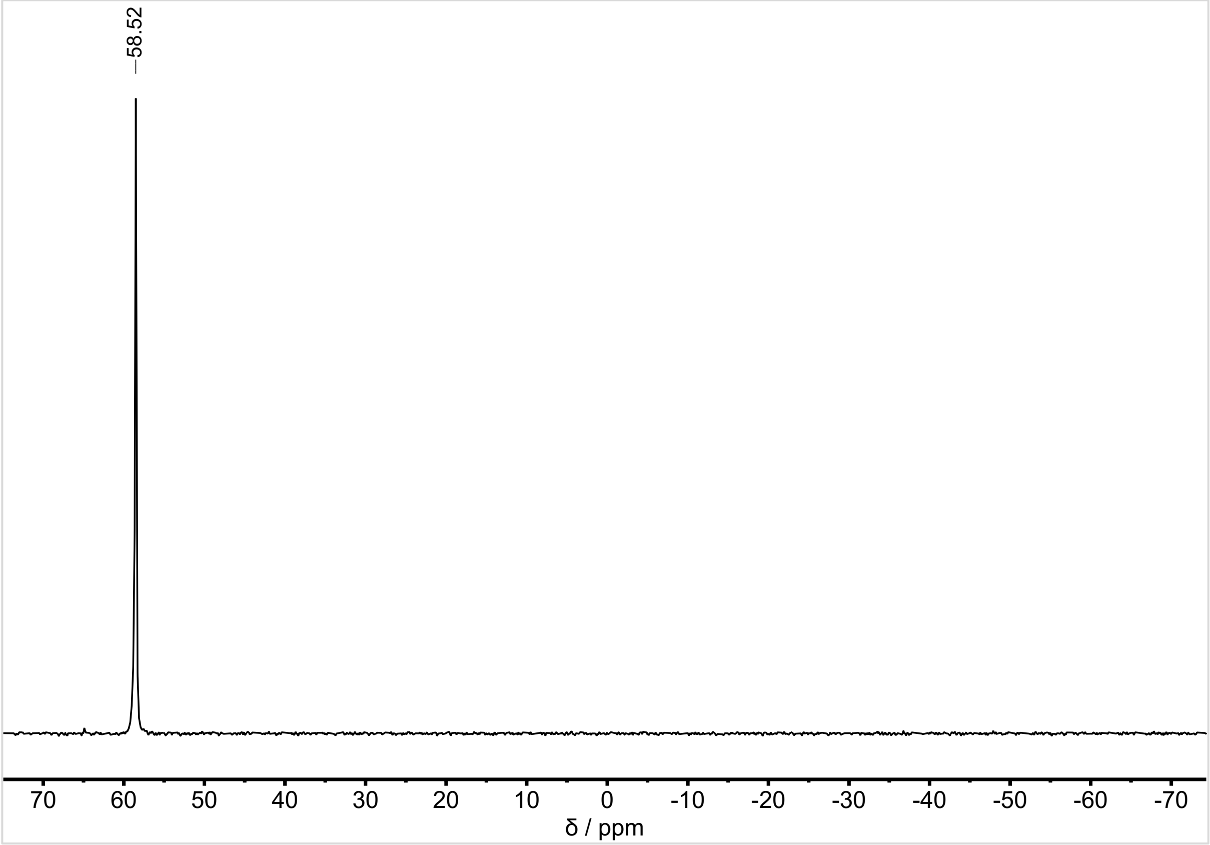

Figure S14: ^{31}P NMR (162 MHz, 1,1,2,2-TCE-d_2) spectrum of **pD*AAAAAAAAAAAAq**.

6. CuAAC Duplex Trapping Experiments

General Procedure for Covalent Trapping Experiments

For each experiment, fresh stock solutions of oligomers, 4-*t*-butylbenzyl azide and $Cu(MeCN)_4PF_6$-TBTA in dry DCM were prepared. The calculated amount from stock solutions of oligomers and 4-*t*-butylbenzyl azide were transferred to a reaction vial and the solvent was removed via evaporation under a stream of N_2. $CuMeCN_4PF_6$-TBTA in dry DCM was then added to the reaction vial under a stream of N_2, and mixtures were left to stir for 48 h at r.t. After reaction completion, the DCM was evaporated under a stream of N_2 and mixtures were re-dissolved in THF/MeOH to a known concentration, then sonicated to ensure that the products were fully dissolved. Mixtures were injected into the LCMS (between 2-5 μL injections) and UV peaks were identified (λ = 254 nm). Peak identity was assigned based on the corresponding mass spectra and retention time. Two different UPLC gradients were used for analysis of product mixtures.

zD*AAAAAAAAAAAAy Covalent Trapping Control Experiments

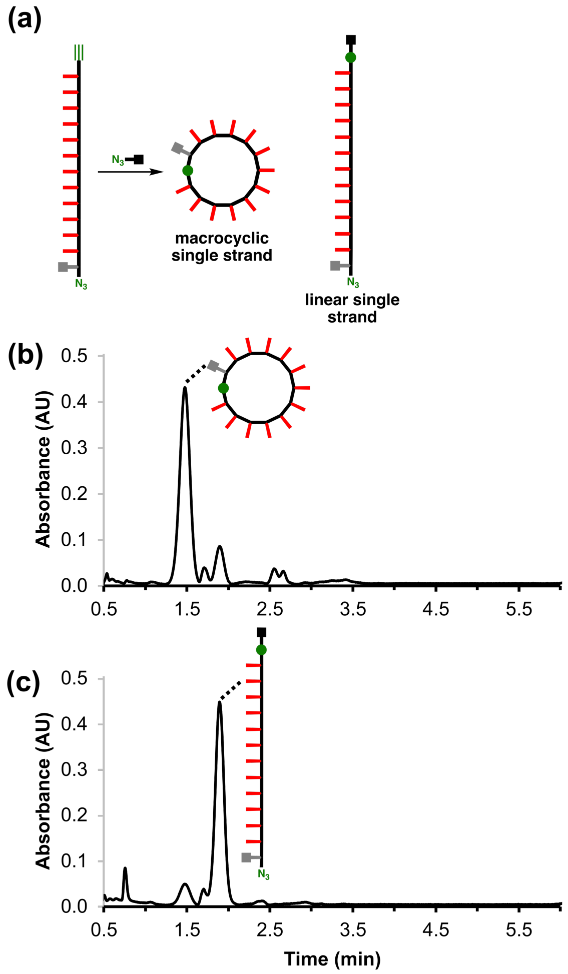

Figure S15: (a) Schematic representation of products formed after CuAAC reaction of **zD*AAAAAAAAAAAAy** in the presence of a competing azide (green circles represent triazoles in the products). **(b)** UPLC trace after reaction of **zD*AAAAAAAAAAAAy** (50 µM), 4-*t*-butylbenzyl azide (100 µM) and $Cu(MeCN)_4PF_6$-TBTA (0.4 mM) in dichloromethane at room temperature for 48 h. **(c)** UPLC trace after reaction of **zD*AAAAAAAAAAAAy** (50 µM), 4-*t*-butylbenzyl azide (5 mM) and $Cu(MeCN)_4PF_6$-TBTA (0.4 mM) in dichloromethane at room temperature for 48 h. *UPLC Conditions:* C4 column at 40 °C using a 55-80% gradient of THF/formic acid (0.1%) in water/formic acid (0.1%) over 5 minutes, then up to 100% THF/formic acid (0.1%) over 1 minute.

7. Fluorescence Experiments

General Procedures for Fluorescence Titrations

Fluorescence spectroscopic data were measured using a Cary Eclipse Fluorescence Spectrophotometer G9800A (Agilent) controlled by Cary Eclipse software. Fluorescence excitation experiments were performed using 10 nm excitation and emission slits, a scan rate of 600 nm/min, a data interval of 1.0 nm, an averaging time of 0.10, and medium PMT voltage at 298 K. Fluorescence emission spectra were recorded using λ_{ex} = 348 nm, and emissions were recorded from λ_{em} = 368-700 nm.

Stock solutions of known concentrations of host (**pDDDDDDDDDDDDf** or a 1:1 mixture of **pDDDDDDDDDDDDf•pD*AAAAAAAAAAAAq**) or guest (DMSO or **pD*AAAAAAAAAAAAq**) were prepared in spectrophotometric grade chloroform. Titration solutions were prepared by dilution of the corresponding stock solutions using spectrophotometric grade chloroform. 2 mL of host solution was added to a fluorescence cuvette and the spectrum was recorded. Aliquots of guest in host solution were added to the cuvette and the spectra were recorded after each addition. The fluorescence intensities were monitored as a function of guest concentration and analysed using *Musketeer* (from GitHub repository, https://github.com/daniilS/Musketeer/releases). See reference:

D. O. Soloviev and C. A. Hunter, *Chem. Sci.*, 2024, **15**, 15299-15310.

Dilution of pDDDDDDDDDDDDf

A solution of **pDDDDDDDDDDDDf** in $CHCl_3$ (10 μM) was titrated into a solution of **pDDDDDDDDDDDDf** in $CHCl_3$ (0.1 μM) at 298 K. Fluorescence emission spectra were taken at λ_{ex} = 348 nm and the intensity of the λ_{em} = 500 nm peak was recorded as a function of **pDDDDDDDDDDDDf** concentration.

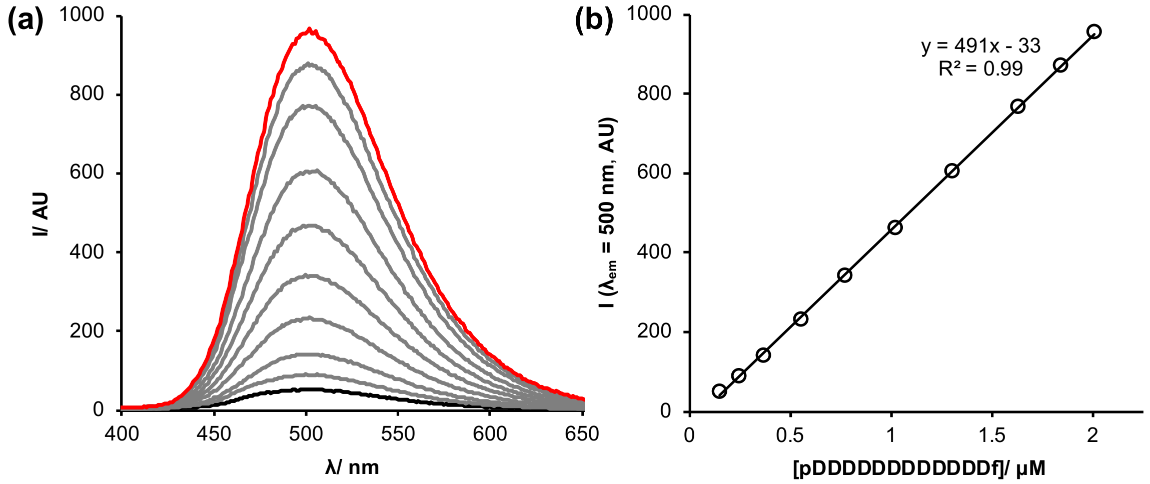

Figure S16: (a) Fluorescence emission spectra (λ_{ex} = 348 nm) for the titration of **pDDDDDDDDDDDDf** (10 μM) into a solution of **pDDDDDDDDDDDDf** (0.1 μM) in chloroform at 298K. **(b)** Plot of the fluorescence intensity (λ_{em} = 500 nm) as a function of **pDDDDDDDDDDDDf** concentration.

Titration of D*AAAAAAAAAAAAq into pDDDDDDDDDDDDfp

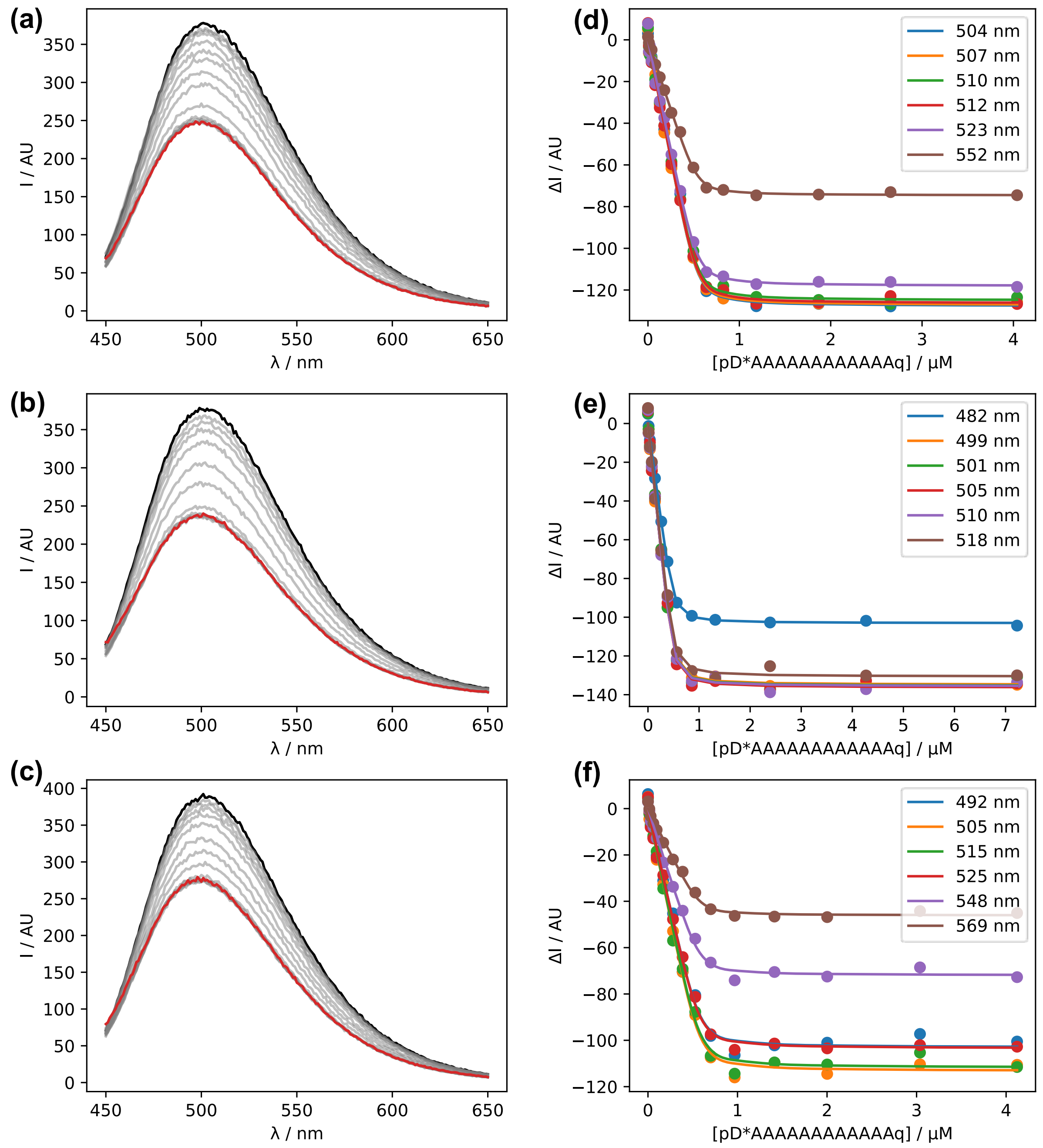

Figure S17: (a)-(c) Fluorescence emission spectra (λ_{ex} = 348 nm) for three titrations of **pD*AAAAAAAAAAAAq** (**(a)** 20.0 μM, **(b)** 25.0 μM, **(c)** 15.0 μM) into **pDDDDDDDDDDDDf** (0.5 μM). The fluorescence spectra of **pDDDDDDDDDDDDf** and the final points of the titrations are shown in black and red respectively. Titrations were carried out in chloroform at 298 K. **(d)-(f)** Change in fluorescence intensity as a function of guest concentration for the three titrations. The lines show the best fits at six different wavelengths to 1:1 binding isotherms allowing for variable host concentration, with K_{duplex} = **(d)** 9.5 × 10^7 M^{-1}; **(e)** 8.3 × 10^7 M^{-1} ; **(f)** 8.2 × 10^7 M^{-1}.

DMSO Denaturation of pDDDDDDDDDDDDf•pD*AAAAAAAAAAAq

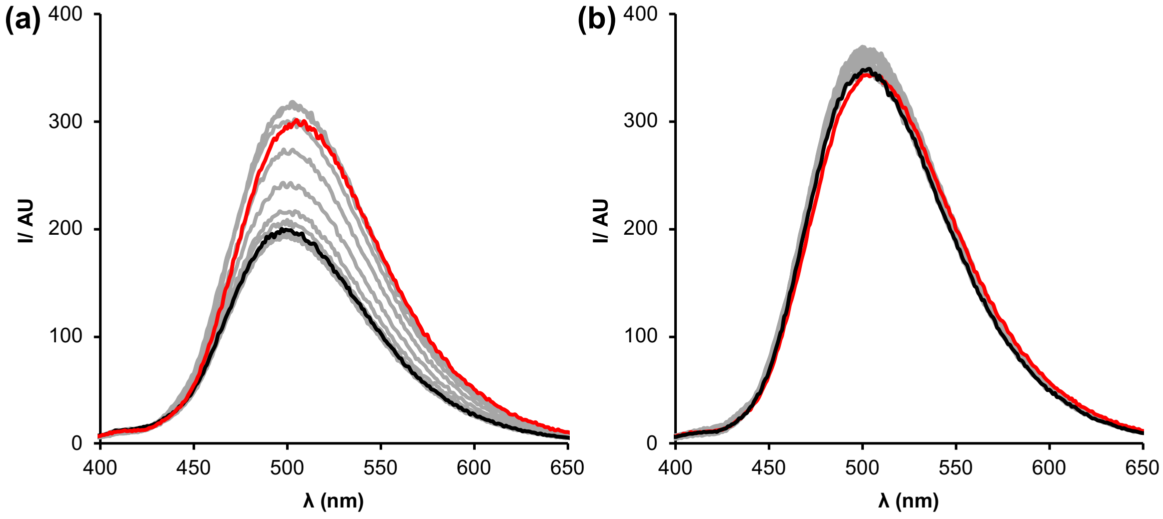

Figure S18: (a) Fluorescence emission spectra (λ_{ex} = 348 nm) for the titration of DMSO (0-650 mM) into a 1:1 mixture of **pDDDDDDDDDDDDf** and **pD*AAAAAAAAAAAq** (1 μM in chloroform at 298K). **(b)** Fluorescence emission spectra (λ_{ex} = 348 nm) for the titration of DMSO (0-650 mM) into **pDDDDDDDDDDDDf** (1 μM in chloroform at 298K). The first and last points of the titrations are shown in black and red respectively.

8. ^{31}P NMR Experiments

NMR Titration of m-cresol into pAp

^{31}P NMR titrations were carried out using a Bruker 500 MHz AVII HD Smart Probe spectrometer. The host (**pAp**) was dissolved in $CDCl_3$ at a known concentration. The guest (m-cresol) was dissolved in the host solution and made to a known concentration. 0.6 mL of host was added to an NMR tube and the spectrum was recorded. Aliquots of guest in host solution were added to the NMR tube, and the spectra were recorded after each addition. The chemical shifts of the host spectra were monitored as a function of guest concentration and analysed using *Musketeer* (available from the GitHub repository, https://github.com/daniilS/Musketeer/releases). The changes in chemical shift were fit to a 1:2 binding isotherm. Errors are quoted as two standard deviations based on three different experiments.

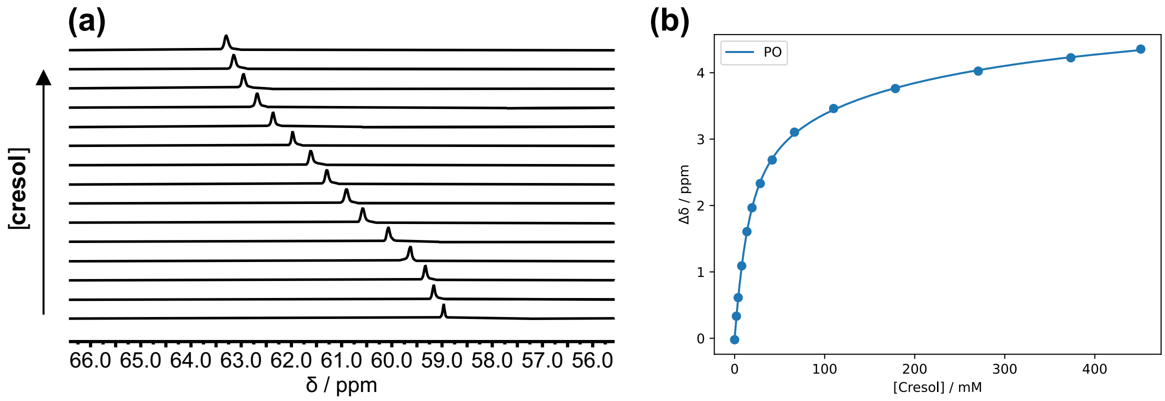

Figure S19: (a) ^{31}P NMR spectra (202 MHz) for the titration of m-cresol into **pAp** (9.6 mM in $CDCl_3$, 298K). **(b)** Plot of the change in chemical shift of the ^{31}P NMR signal as a function of guest concentration, where the line represents the best fit to a 1:2 binding isotherm with $K_1 = 120 \pm 20$ M^{-1}, $K_2 = 4 \pm 1$ M^{-1}, $\delta_{free} = 58.9$ ppm, $\delta_{bound,\,1} = 62.0$ ppm and $\delta_{bound,\,2} = 64.1$ ppm.

DMSO Denaturation of zD*AAAAAAy•zDDDDDDy

For full details of the denaturation experiment see:

M. Dhiman, R. Cons, O. N. Evans, J. T. Smith, C. J. Anderson, R. Cabot, D. O. Soloviev, and C. A. Hunter, *J. Am. Chem. Soc.*, 2024, **146**, 9236–9334.

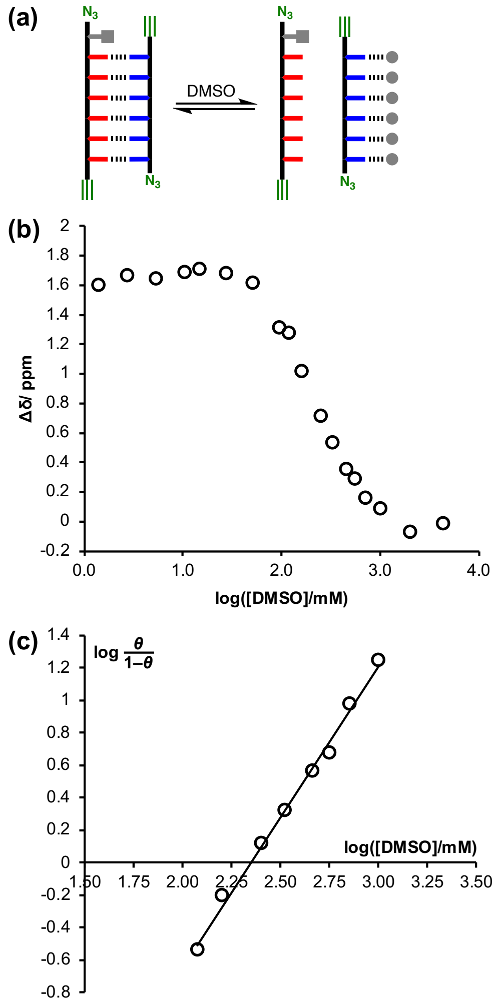

Figure S20: (a) DMSO denaturation of the **zD*AAAAAAy•zDDDDDDy** duplex (DMSO shown as grey circles). **(b)** Complexation-induced change in ^{31}P NMR chemical shift (Δδ) for duplex denaturation plotted as a function of DMSO-d_6 concentration in 1,1,2,2-tetrachloroethane-d_2 at 298 K (Δδ is the difference between the chemical shift of a 1:1 mixture of **zD*AAAAAAy•zDDDDDDy** and pure **zD*AAAAAAy** at the same concentration of DMSO). **(c)** Hill plot for the denaturation experiment (θ = (1.65-Δδ)/1.65) showing the best fit straight line, y = 1.9 x – 4.3.
